# Genome-Wide Analyses of MicroRNA Profiling in Interleukin-27 Treated Monocyte-Derived Human Dendritic Cells Using Deep Sequencing: A Pilot Study

**DOI:** 10.3390/ijms18050925

**Published:** 2017-04-28

**Authors:** Xiaojun Hu, Qian Chen, Bharatwaj Sowrirajan, Marjorie Bosche, Tomozumi Imamichi, Brad T. Sherman

**Affiliations:** Laboratory of Human Retrovirology and Immunoinformatics, Applied and Developmental Research Directorate, Leidos Biomedical Research, Inc., Frederick National Laboratory for Cancer Research, Frederick, MD 21702, USA; hux4@mail.nih.gov (X.H.); chenq3@mail.nih.gov (Q.C.); bharatwaj.sowrirajan@nih.gov (B.S.); mbosche@mail.nih.gov (M.B.); bsherman@mail.nih.gov (B.T.S.)

**Keywords:** microRNA, IL-27, dendritic cells, deep sequencing

## Abstract

MicroRNAs (miRNAs) regulate gene expression and thereby influence cell fate and function. Recent studies suggest that an abundant class of miRNAs play important roles in immune cells, such as T cells, natural killer (NK) cells, B cells, and dendritic cells (DCs). Interleukin (IL)-27 is a member of the IL-12 family of cytokines with broad anti-viral effects. It is a potent inhibitor of HIV-1 infection in CD4+ T cells and macrophages, as well as monocyte-derived immature dendritic cells (iDCs). This pilot study compared miRNA profiles between iDCs and IL-27-treated iDCs (27DCs) using deep sequencing methods and identified 46 known miRNAs that were significantly differentially expressed in 27DCs: 36 were upregulated and 10 downregulated by IL-27. Many of the potential target genes of these miRNAs are involved in IL-27 associated pathways, such as JAK/STAT, MAPKs, and PI3K and several were also previously reported to be involved in the regulation of human DC function. This study found that these miRNAs also potentially target several viral genomes and therefore may have antiviral effects. Four of these differential miRNAs (miR-99a-5p, miR-222-3p, miR-138-5p, and miR-125b-5p) were validated using quantitative reverse transcription PCR (RT-qPCR). Twenty-two novel miRNAs were discovered from deep sequencing and confirmed using RT-qPCR. This study furthers the understanding of the role of IL-27 in immunity and lays a foundation for future characterization of the role of specific miRNAs in DCs.

## 1. Introduction

Dendritic cells (DCs) are a type of antigen-presenting cell (APC), which are crucial for the regulation of adaptive and innate immunity [1]. They present peptide antigens via HLA molecules to T cells in lymph nodes and provide activating stimuli to these cells as initiators of the adaptive immune response [2]. DCs also respond to invading pathogens and induce interferon or other cytokines via pattern recognition receptors as part of the innate immune response. They mediate the spread of HIV to CD4+ T cells in lymphoid tissues in vivo and DC-HIV interactions play an important role for DCs in HIV transmission at mucosal surfaces and in viral pathogenesis [3]. DCs are also one of the cell types potentially serving as an HIV reservoir [4].

MicroRNAs (miRNAs) are fragments of non-coding RNA, approximately 19–25 nucleotides in length, and derived from an approximately 70–120 nucleotide hairpin precursor molecule in intergenic chromosomal regions or within introns of protein coding genes [5]. They post-transcriptionally regulate gene expression by binding to partially complementary sequences mostly in the 3′UTR of mRNAs [6]. They govern hundreds and even thousands of genes and could be important therapeutic targets and promising biomarkers [7,8]. Many miRNAs are selectively expressed in cells of the innate and adaptive immune system, such as T cell subsets, natural killer (NK) cells, and B cells [9]. They are vital for cell differentiation and homeostasis, interactions with pathogens, cytokine response, and induction of tolerance [10]. miRNAs regulate DC differentiation and function [9], and exosome-shuttle miRNAs likely mediate DC-to-DC interactions [11]. miRNAs are also transferred from T cells to APCs [12], and HIV infectivity has been reported to be influenced by cellular miRNAs that modulate HIV-1 infection and replication [13].

Interleukin (IL)-27, a member of the IL-12 cytokine family (IL-12, IL-23, IL-27, and IL-35), is recognized as both a pro- and anti-inflammatory cytokine that mediates both innate and adaptive immune responses [14]. It is largely secreted from activated APCs including macrophages and DCs, and is composed of two subunits: p28 and Epstein-Barr virus-induced gene 3 (EBI-3) [15]. IL-27 mediates signaling through members of the Janus kinase/signal transducer and activator of transcription (JAK/STAT) pathway, and STAT-1, -3, and -5 are especially activated following engagement of IL-27 with its receptor [16,17,18,19]. Moreover, we have previously reported that IL-27 induces the TGF-β activated kinase 1 (TAK1) mediated signaling pathway [20]. Recent studies demonstrate that IL-27 activates the mitogen-activated protein kinase (MAPK)/ERK pathway [21,22] as well as the phosphatidylinositol 3-kinase (PI3K)/AKT signaling pathway [23].

The authors of the present study previously reported that IL-27 inhibits HIV-1 replication in CD4+ T cells [24] and macrophages [20]. Recently, it was demonstrated that IL-27 is a potent inhibitor of HIV-1 replication in monocyte-derived immature DCs (iDCs) [19] and is also a potent regulator of reactive oxygen species induction in iDCs [25]. To provide better insight of IL-27 antiviral function in iDCs and to detect cellular miRNAs that might be involved in this function, miRNA-specific deep sequencing in iDCs and IL-27-treated immature DCs (27DCs) were performed to identify differentially expressed known miRNAs and discover novel miRNAs.

## 2. Results

### 2.1. miRNA Library Analysis

Illumina deep sequencing produced between 49 and 69 million reads (2500 and 3500 Mbases) per sample (Table S1). After quality trimming (Phred score ≥30) and length filtering (17–35 bp), 38% to 53% of the reads were kept as “clean” reads, with a range of 21.2 to 33.4 million reads per sample. A very high percentage of these “clean” reads were mapped to the human genome (hg38) with all samples having over 95% of the associated reads mapped. Length distribution analysis showed that one single prominent peak was found at 23 nucleotides (Figure S1) for all samples. This further supported the dominant presence of miRNAs in all libraries. There was also a small peak at 31 nucleotides, which was compatible with Piwi-interacting RNAs (piRNAs). This suggests that some piRNAs may be present in dendritic cells.

### 2.2. Highly Expressed miRNAs in DCs

**Figure 1:**
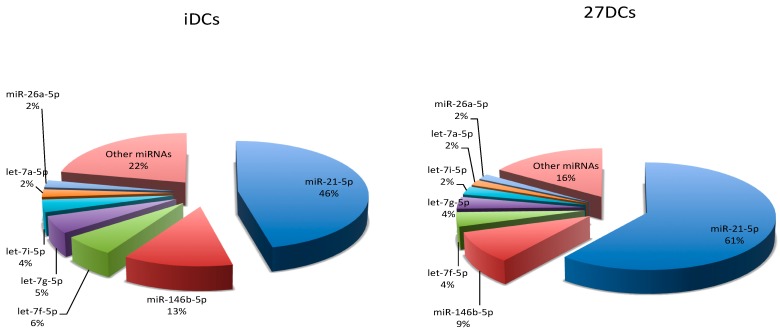
Top seven most abundant microRNAs (miRNAs) in dendritic cells. The average proportion of the top seven most abundant miRNA reads relative to the total number of mature miRNA in immature dendritic cells (iDCs) and IL-27-treated iDCs (27DCs).

miRNA raw read counts were normalized by count-per-million (CPM) using EdgeR [26]. The relative number of sequence reads for each miRNA (percentage in the sample) indicated their abundance in each sample. Seven miRNAs (miR-21-5p, miR-146b-5p, let-7f-5p, let-7g-5p, let-7i-5p, let-7a-5p, and miR-26a-5p) that were highly expressed are shown in Figure 1. Their abundance accounted for 78% and 84% of the total miRNA reads in iDCs and 27DCs, respectively. Interestingly, miR-21-5p represented around 50% of the total miRNA pool in both iDCs and 27DCs. Its expression in 27DCs was 14% higher than in iDCs. The expression of miR-146b-5p, let-7g-5p, let-7f-5p, and let-7i-5p was down-regulated by IL-27 (not significantly).

### 2.3. Differential miRNA Expression in 27DCs

**Figure 2:**
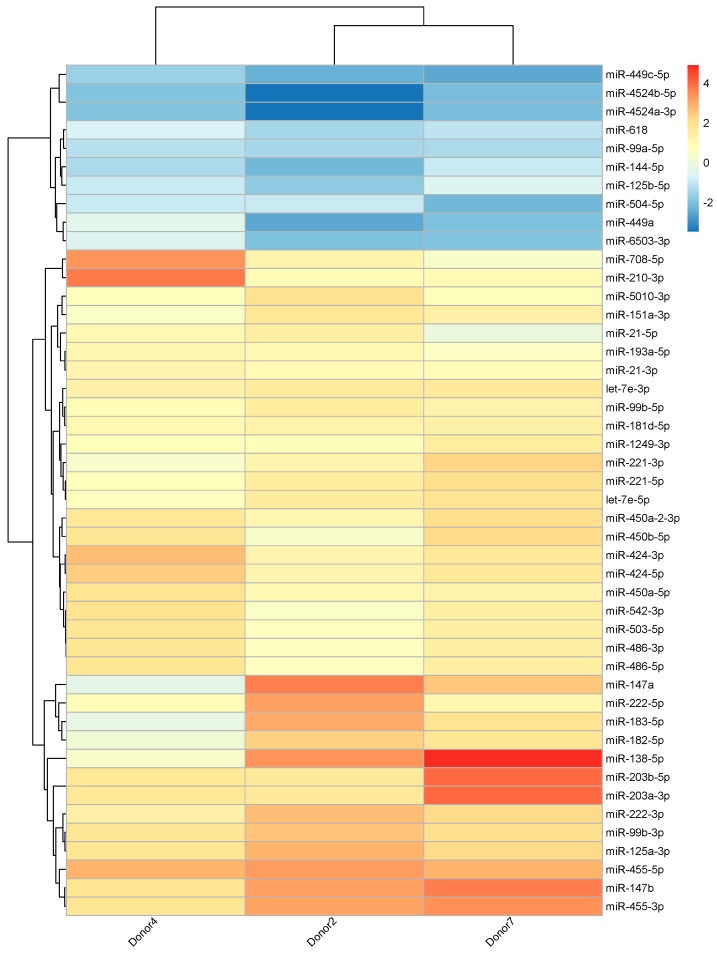
miRNA expression profile for IL-27 regulated known miRNAs. Heatmap shows heterogeneity between samples. Values are 27DC vs iDC log2 fold change for each donor. The color scale shown at the right illustrates the relative expression level of the indicated miRNA in each sample: blue denotes down-regulated (log2 fold change < 0) and red denotes up-regulated (log2 fold change > 0).

A total of 46 known miRNAs were significantly differentially expressed between iDCs and 27DCs (Figure 2) based on the sequencing data. Volcano and MA plots (Figures S2 and S3) further show these miRNAs’ expression and significance. Thirty-six miRNAs were significantly up-regulated by IL-27 in DCs and 10 were significantly down-regulated. Seventeen miRNAs had high abundance (>200 normalized reads) in at least one sample. Among them, 12 miRNAs (let-7e-5p, miR-151a-3p, miR-21-5p/-3p, miR-221-5p/-3p, miR-222-3p, miR-424-3p, miR-450a-5p, miR-450b-5p, miR-503-5p, and miR-99b-5p) were up-regulated by IL-27 and two miRNAs (miR-99a-5p and miR-125b-5p) were down-regulated.

### 2.4. KEGG Pathway Enrichment Analysis

A single miRNA may have many target genes and a single gene can be regulated by several miRNAs. To determine which genes might be modified by the miRNAs identified in the present study, miRNA functional enrichment analyses was performed using the DIANA-miRPath (v3.0) online tool [27]. Unlike TargetScan [28] using 3′UTR of an mRNA, the DIANA-microT-CDS prediction algorithm searches all possible miRNA seed binding sites both in the coding sequence and in the 3′UTR of a gene. The significant target gene enriched KEGG pathways are reported based on a Fisher’s Exact Test. Forty-six differential miRNAs were analyzed by DIANA-miRPath. Fifty-five significant KEGG pathways (*p*-values < 0.05) were reported. Splitting the miRNAs of interest into up- and down-regulated groups for analysis yielded similar results.

The DIANA-miRPath results revealed that IL-27-related miRNAs and their predicted targets may function in pathways associated with immune activation (Table 1). These include ErbB, MAPK, PI3K/AKT, and TGF-β signaling pathways. This was consistent with previous reports that IL-27 regulates both innate and adaptive immunity largely via JAK/STAT [17,29,30,31], MAPK, and PI3K/AKT signaling pathways [32]. Although JAK/STAT signaling pathway was not enriched by DIANA-miRPath tool, we found that 35 out of 46 miRNAs were involved in this pathway. These included let-7e-5p, miR-21-5p/-3p, miR-221-5p/-3p, miR-424-5p/-3p, miR-450a-5p, miR-450b-5p, miR-503-5p, etc. STAT1 is targeted by miR-5010-3p and STAT3 is targeted by miR-21-5p, miR-125b-5p, and miR-4524a-3p. JAK2 is targeted by miR-221-5p, and JAK3 is targeted by miR-221-3p and miR-222-3p.

### 2.5. Validation of Differential miRNAs by RT-qPCR

**Figure 3:**
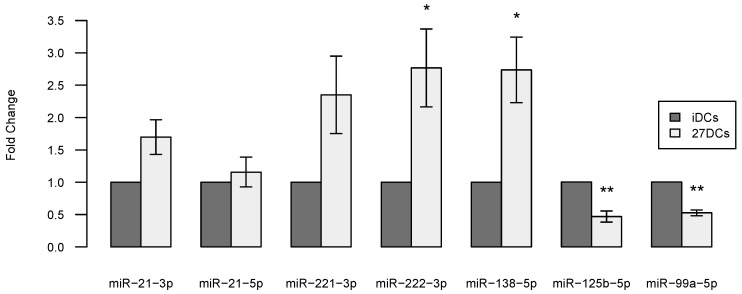
RT-qPCR validation of seven differential miRNAs in three different donors (Donor 22, 24, and 27). The expression level of each miRNA was normalized to RNU44 and converted to fold change (27DCs vs. iDCs). miRNA expression in 27DCs (gray) relative to iDCs (black) for seven miRNAs are shown. Error bars represent standard error (SE). * denotes *p*-values < 0.05 and ** denotes *p*-values < 0.01 for differences in miRNA expression between iDCs and 27DCs. A two-tailed *t*-test was used to calculate the *p*-values.

Seven out of the 46 differentially expressed known miRNAs were selected for RT-qPCR validation, each of which have potential target genes in all five enriched pathways from the KEGG pathway enrichment analysis (see Table 1) and also play roles in DCs (see discussion). Traditional Taqman miRNA assays were conducted using commercial kits with three independent donors for validating the seven miRNAs. The RT-qPCR results for the seven miRNAs exhibit the same trend as the sequencing results. Two of the upregulated miRNAs (miR-222-3p, miR-138-5p) and two of the downregulated miRNAs (miR-125b-5p, miR-99a-5p) were confirmed to be significantly differentially expressed between iDCs and 27DCs (Figure 3). miR-21-5p was highly expressed and significantly regulated by IL-27 based on the sequencing data but was not confirmed by RT-qPCR. The significance of the expression of miR-21-3p and miR-221-3p were also not confirmed by RT-qPCR (*p* = 0.060 and *p* = 0.087, respectively) perhaps due to sample size limitations of the study.

### 2.6. Potential Viruses Targeted by Differential miRNAs

IL-27 can inhibit replication of many viruses [33,34], and cellular miRNAs may have direct antiviral functions [35]. For the 46 differential miRNAs, potential target sites in 745 viral reference genomes from NCBI were detected using miRanda [36]. As shown in Table S2, the forty-six miRNAs targeted 165 human viruses including human herpesvirus, hepatitis B virus, hepatitis C virus, influenza, ebola, west nile virus, etc. Interestingly, the relatively highly expressed miRNAs (miR-21-5p, miR-21-3p, miR-221-3p, miR-222-3p, let-7e-5p, miR-99a-5p, and let-7c-5p) targeted Human herpesvirus and other viruses.

### 2.7. Novel miRNA Discovery

Novel miRNA discovery was performed using miRDeep2 [37]. After analyzing a total of six samples (iDC and 27-DC for each of three donors), 183–302 novel miRNA candidates per sample with some donor dependency were initially found. After merging and removing duplicates, a total of 739 novel miRNA candidates were retained and further filtered with the following criteria: (1) precursor minimum free energy (MFE) ≤−20 kcal/mol; (2) mature GC content ≤80%; (3) not another RNA species (tRNA, rRNA, scaRNA, snoRNA, snRNA, Y-RNA, etc.) or a known miRNA; (4) not a duplicate precursor; and (5) maximum mature read count ≥2. After filtering, 361 novel miRNA candidates were retained (Table S3). Of these, 311 novel miRNA candidates had lower maximum read counts (2–50), with the remaining 50 having maximum read counts >50. Considering miRNA expression level (high, middle, or low), differential significance among donors, and encoding genes associated with immune responses, 22 candidates were selected for RT-qPCR confirmation (Table 2). All novel miRNA secondary structures and the aligned read profiles are shown in Figure S4 in Supplementary Materials. Of the 22 candidates, four novel miRNAs (QXBT3, 10, 15, 16) were significantly differentially regulated by IL-27 and QXBT13 had a relatively high abundance (maximum read count of 18285). To test the lower detection limit of miRNA-Seq, 14 of the 22 candidates had maximum read counts below 50.

### 2.8. Validation of Novel miRNAs

**Figure 4:**
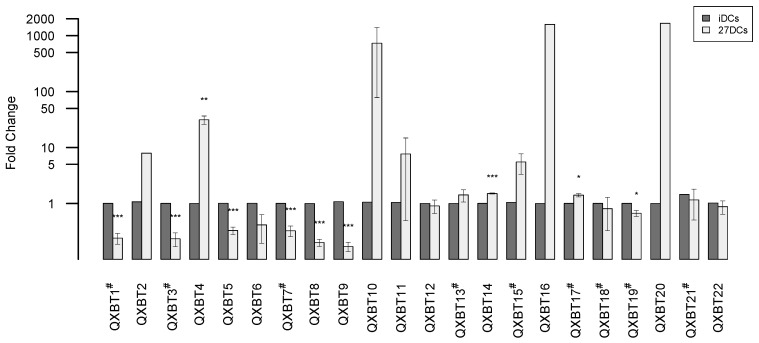
RT-qPCR validation of novel miRNAs in three different donors (Donor 12, 14, and 17). The expression level of each miRNA was normalized to a small RNA reference and converted to fold change (27DCs vs. iDCs). miRNA expression in 27DCs (gray) relative to iDCs (black) for miRNAs are shown. Error bars represent standard error (SE). *Y* axis is log scale fold change. # in the miRNA name denotes sample from the traditional RT-qPCR assay; the others from the advanced assay. * above the bar denotes *p*-values < 0.05, ** denotes *p*-values < 0.01 and *** denotes *p*-values < 0.001. A two-tailed *t*-test was used to calculate the *p*-values.

To validate the expression levels of the novel miRNA candidates, advanced and traditional Taqman miRNA RT-qPCR assays were performed on three independent donors. In the advanced assay, a universal RT primer is used to generate cDNA while, in the traditional assay, a gene-specific RT primer (Figure S5) is used. Therefore, the traditional assay may have a higher sensitivity than the advanced assay. The advanced assay was used initially, but after some samples failed, tests were repeated with the traditional assay. Finally, 13 out of 22 miRNAs were successfully confirmed with the advanced assay, while the other nine miRNAs were successfully confirmed with the traditional assay (Figure 4). From the RT-qPCR data, three of the novel miRNAs were significantly up-regulated in 27DCs and seven were significantly down-regulated in 27DCs. The *t*-test results for QXBT10 and QXBT15 were not significant due to a large range of variability between donors. Some novel miRNAs (QXBT2, QXBT16, and QXBT20) were not found in all samples using the advanced method. This could be a method sensitivity issue or potentially, the miRNA expression was donor dependent. Interestingly, two miRNAs (QXBT16 and QXBT17) with very low read counts (maximum read count of six and two, respectively) were confirmed.

## 3. Discussion

This pilot study quantified the expression of 1241 known miRNAs (miRBase v21) in iDCs using next-generation sequencing (NGS) technology. The result from the NGS assay demonstrated that 46 of these miRNAs were significantly differentially up- or down-regulated by IL-27. Among them, four miRNAs (miR-125b-5p, miR-138-5p, miR-222-3p, and miR-99a-5p), which potentially target genes involved in ErbB, Wnt, TGF-β, MAPK, and PI3K signaling pathways, were validated by RT-qPCR. In addition, 22 novel miRNAs were discovered using NGS data and confirmed by RT-qPCR. Although most of the novel miRNAs had very low expression, they all were confirmed, including one novel miRNA with only two reads. While the small sample size is a limitation for this study, the information derived lays a foundation to further our understanding of the role of IL-27 and miRNAs in DCs.

Seven known miRNAs—miR-21-5p, miR-146b-5p, let-7f-5p, let-7g-5p, let-7i-5p, let-7a-5p, and miR-26a-5p—were highly expressed, and the expression of those miRNAs contributed 78% and 86% of the total miRNAs in iDCs and 27DCs, respectively. miR-146b has been reported to regulate DC apoptosis [38] and let-7i has been demonstrated to regulate DC maturation through targeting suppressor of cytokine signaling 1 (SOCS1) [39]. Of these miRNAs, the most abundantly expressed was miR-21-5p, which accounted for 46% of the miRNA pool in iDCs and 64% of the pool in 27DCs. miR-21 (miR-21-5p/3p) is one of the most abundantly expressed miRNAs in many cancer cell types and has been relatively well studied [40,41]. It is expressed in hematopoietic cells of the immune system, particularly monocytes, macrophages, DCs, and T cells. Its expression is further enhanced in many diseased tissues and in particular, inflammation-associated diseases. It may be a key switch in the inflammatory response and play a role in regulating the balance of pro- and anti-inflammatory cytokine responses [41]. Based on the sequencing result, IL-27 significantly increased miR-21-5p/-3p expression in DCs, suggesting that miR-21 may be involved in IL-27 mediated pro-inflammatory and anti-inflammatory roles. However, significant differential expression of miR-21-5p/-3p was not confirmed by RT-qPCR perhaps due to the sample size limitation in this study. Further study is needed to understand the relationship between IL-27 and miR-21.

miRNAs can regulate DC differentiation and function, such as their maturation process, antigen presentation capacity and cytokine release [9,42]. It is reported that miR-21, let-7e, miR-99b, and miR-125a are coordinately up-regulated during iDC differentiation through regulation of WNT1 and JAG1 genes in the Notch/Wnt signaling pathway [43]. Another report demonstrated that miR-221 is up-regulated in immature DCs upon differentiation from monocytes, resulting in down-regulation of p27kip1 in immature DCs, and thereby contributing to immature DC homeostasis [44]. p27kip1, a cell cycle regulator, directly regulates DC apoptosis [45]. Moreover, p27kip1 is one of the targets of the miR-221-222 cluster [46]. In addition, both miR-221 and miR-222 influence DC subset differentiation [47]. In the present study, the aforementioned miRNAs (miR-21-3p/-5p, let-7e-3p/-5p, miR-99b-3p/-5p, miR-125a-3p, miR-221-3p/-5p, and miR-222-3p/-5p) affecting DC functions were differentially regulated by IL-27 based on the sequencing results, suggesting that they may play a role in IL-27 regulation of DC function.

IL-27 is largely secreted from activated APCs such as macrophages and DCs upon stimulation. It regulates both innate and adaptive immune responses largely via the JAK/STAT signaling pathway [17,18,48]. In our study, we found that the potential targets of 35 out of 46 differential miRNAs were involved in the JAK/STAT signaling pathway. It has been reported that IL-27 signaling stimulates MAPK/ERK [21,22] and PI3K/AKT signaling pathways [23] which were found to be enriched in our KEGG pathway analysis. This demonstrates that miRNAs may be involved in networks of IL-27 regulated immunity.

Recent studies have shown that IL-27 can inhibit replication of viruses, such as influenza [49], hepatitis C [50], and HIV-1 [19,20]. One antiviral defense mechanism is for miRNAs to repress the gene expression levels of the virus [51]. A previous study observed that IL-27 could induce antiviral miRNAs in macrophages [34]. In this study, it was found that all 46 differential miRNAs in DCs regulated by IL-27 had binding sites in a number of viral genomes, suggesting that miRNAs may play an important role in the antiviral activity of IL-27. From these results, miR-21-5p and miR-138-5p potentially target hepatitis C and influenza A virus, respectively. This finding is consistent with the previous reports that IL-27 represses hepatitis C [50] and influenza A [49]. Of note, no targets were identified in the HIV-1 reference (NC_001802.1) suggesting that the anti-HIV properties of IL-27 may be mediated via modifications in the host cell factors rather than direct anti-viral effects of IL-27 induced miRNAs. Currently, the mechanism of the anti-HIV effect in 27DCs is under study.

The identification of novel miRNAs was validated using both advanced and traditional Taqman RT-qPCR methods. Thirteen miRNAs were confirmed by the advanced method and nine were confirmed by the traditional method. The differences between the two methods are the cDNA primers and the internal controls. The advanced assay uses a universal RT primer and hsa-miR-423-5p as a control. The traditional assay uses miRNA-specific RT primers and RNU44 as a control. In the RT-qPCR method, prior to the RT primer annealing, the total RNA is ligated with an adaptor encoding the complement sequence of the RT primer. Efficiency of the adaptor ligation does affect the RT primer annealing. The traditional method uses miRNA-specific RT primers leading to better annealing to the targeted miRNA as opposed to the universal RT primer used in the advanced method. In our study, nine novel miRNAs were not detected by the advanced assay but were confirmed by the traditional method, suggesting that the traditional method is more sensitive than the advanced method. Some miRNAs were not detected in all donors using the advanced method, which may be due to the method sensitivity.

## 4. Materials and Methods

### 4.1. Generation of DC Subtypes

CD14+ monocytes were isolated from peripheral blood enriched leukopacks (Blood Bank, National Institute of Health, Bethesda, MD, USA) from donors using MACS CD14 MicroBeads (Miltenyi Biotec, Auburn, CA, USA). Monocyte-derived immature DCs (iDCs) were generated by incubating 0.5 × 10^6^/mL monocytes in G4 medium (RPMI 1640 containing 10% fetal bovine serum (Hyclone, UT, USA), 2 mM glutamine, 25 mM HEPES (*N*-2-hydroxyethylpiperazine-N9-2-ethanesulfonic acid) (Quality Biological, Gaithersburg, MD, USA), 10 mg/mL gentamicin, 50 ng/mL GM-CSF (R&D Systems, Minneapolis, MN, USA) and 50 ng/mL IL-4 (R&D Systems) at 37 °C in a CO_2_ (5%)) incubator for seven days. The IL-27 treated DCs (27DCs) were induced from monocytes cultured in G4 medium (iDCs (1 × 10^6^) stimulated) with IL-27 (100 ng/mL) at 37 °C for seven days. The culture media was changed with fresh G4 media every 3–4 days. In this study, we used a total of nine donors. Donor 2, 4, and 7 were used for sequencing; Donor 12, 14, and 17 were used for the validation of 22 novel miRNAs; Donor 22, 24, and 27 were used for the validation of seven differential miRNAs.

### 4.2. miRNA Library Preparation and Sequencing

Total RNA was extracted with chloroform and phenol. Prior to RNA sequencing, samples were subjected to analysis by the Agilent Bioanalyzer RNA Nano chip and the small RNA chip to confirm RNA purity and quality (Santa Clara, CA, USA). All samples had an RNA integrity number of 10. Six microRNA libraries were prepared using the NEBNext Multiplex Small RNA Library Prep for Illumina protocol (New England BioLabs, Ipswich, MA, USA) according to the manufacturer’s instructions and sequenced on the Hiseq 2500 using v4 chemistry for single end sequencing (San Diego, CA, USA). Ninety-four percent or more of the bases for all samples had a Phred quality score of Q30 or greater and all samples yielded between 49 and 68 million reads. All small RNA sequences have been deposited into NCBI SRA database under accession number: SRX2406023.

### 4.3. Differential Expression Analysis

Cutadapt [52] was used to remove adaptors and trim reads with a Phred quality score threshold of 30 (Q30) and length threshold of 17–35 bp producing a set of “clean” reads for each sample. The “clean” reads were mapped to the human reference genome hg38 using the Burrows–Wheeler aligner, BWA (v0.7.10-r789) [53] with one mismatch as recommended by others [54]. Known miRNA read counts were determined by bedtools multicov [55] with miRBase (v21) [56]. The read count matrix was analyzed using the edgeR package 3.16.5 [57] in R version 3.2.3, where general linear model (GLM) was designed with donors+treatment for paired sample comparisons. Resulting *p*-values were corrected with Benjamini and Yekutieli correction. The significant miRNAs were selected based on fold change >1.5 or <−1.5, false discovery rate (FDR) <0.05 and at least one sample count (maximum read count) >50. A heat map was generated by pheatmap 1.0.8 [58] using log2 fold change from log2 counts per million (logCPM) by edgeR.

### 4.4. KEGG Pathway and GO Term Enrichment Analysis

The Kyoto Encyclopedia of Genes and Genomes (KEGG) Pathway [59] enrichment analysis was performed by DIANA miRPath v.3. This calculates enrichment based on miRNA predicted target genes derived from DIANA-microT-CDS [60]. The selected 46 differential miRNAs were used for an enrichment analysis. The IL-27 related pathways with *p*-values < 0.05 were reported as significant in this study.

### 4.5. Prediction of Viruses Targeted by miRNAs

745 human viral reference genome sequences were downloaded from the NCBI viral genome browser (available online: http://www.ncbi.nlm.nih.gov/genomes/GenomesGroup.cgi?taxid=10239&opt=Virus&sort=genome) and were scanned by miRanda [36] with a minimum free energy <−20 kcal/mol using 46 differential miRNAs. The viral binding sites were further filtered with a pairing score ≥100 as potential miRNA targets.

### 4.6. Discovery of Candidate Novel miRNAs

miRNA-Seq “clean” reads were collapsed and then mapped to the human reference genome hg38 using BWA with one mismatch. Mapped reads were analyzed using the miRDeep2 algorithm [37] for generating novel miRNAs candidates. A list of the candidates from different samples were merged using their genomic coordinates with bedtools intersect [55]. For duplicate candidates, the region with the highest mature count was kept. To eliminate other RNAs, like snoRNA, snRNA, tRNA, rRNA, Y-RNA, etc., the region was annotated by GENCODE v24 (available online: www.gencodegenes.org) and ANNOVAR [61] with hg38, and the precursors were loaded into RFam (v12.1, available online: http://rfam.xfam.org/) for characterization. The precursor minimum free energy (MFE) was computed by RNAFOLD [62] and the mature sequence GC content was calculated with an in-house Perl script. The interesting candidates were further identified by their coordinates with tracks supplied by the UCSC genome browser (available online: https://genome.ucsc.edu/) and their RNA secondary structures from miRDeep2. The mature sequence coordinates were derived from the precursor coordinates with an in-house Perl script, and read count and differential novel miRNAs were determined based on the previously defined steps for the known miRNAs. Maximum read count was defined as the maximum count across all 6 samples.

### 4.7. Quantitative RT-PCR for miRNA Validation

To quantitate the relative expression of the novel miRNAs, quantitative real-time PCR was performed using the TaqMan Advanced miRNA Assays (Thermo Fisher Scientific, Waltham, MA, USA) with the iCycler real-time PCR detection system (Bio-Rad, Hercules, CA, USA) following the manufacturer’s instructions. hsa-miR-423-5p was used as an endogenous control as recommended by the manufacturer. Since the advanced method uses the universal RT primer for cDNA synthesis, some miRNAs were not detected. Traditional TaqMan MicroRNA Assays (Thermo Fisher Scientific) were performed for those miRNAs that were not detected using the advanced method with RNU44 used as an endogenous control. The traditional assay was also used for differential miRNA validation. Gene-specific primers and probes were custom made by Thermo Fisher Scientific (Waltham, MA, USA). The relative expression of each miRNA was calculated using the 2^−ΔΔ*C*t^ (cycle threshold, *C*_t_) method [63]. The significance between iDCs and 27DCs for each miRNA was analyzed using a two-tailed *t*-test in R with iDC values normalized to 1.

## 5. Conclusions

Monocyte-derived DCs play a critical role in the development of an immune response. IL-27 can modulate the immune response through its effect on iDCs. The present study demonstrated that miRNAs may mediate some of the effects of IL-27 on DCs. Forty-six miRNAs were regulated by IL-27 based on the sequencing results and potentially target genes in the IL-27 related pathways: JAK/STAT, MAPKs, and PI3K. Four of these were confirmed by RT-qPCR (miR-99a-5p, miR-222-3p, miR-138-5p, and miR-125b-5p). In addition, 22 novel miRNAs were discovered and confirmed. While this study is considered a pilot study due to sample size limitations, the results provide a resource for the understanding of IL-27 mediated immunity and lay a foundation for the future characterization of the role of specific miRNAs in the regulation of DCs.

## Figures and Tables

**Table 1:** Kyoto Encyclopedia of Genes and Genomes (KEGG) pathway analysis of the target genes of differential miRNAs regulated by IL-27.

KEGG Pathway	*p*-Values	Gene Count	miRNA Count
ErbB signaling pathway	8.98 × 10^−6^	58	35
Wnt signaling pathway	4.60 × 10^−5^	84	36
TGF-β signaling pathway	4.99 × 10^−5^	50	33
MAPK signaling pathway	1.30 × 10^−3^	143	38
PI3K-Akt signaling pathway	1.60 × 10^−2^	177	41

**Table 2:** Sequence and genomic location of novel miRNAs.

Novel miRNA	GenBank Accession Number	Mature Sequence	Precursor Genomic Location (hg38)	Precursor Minimum Free Energy (kcal/mol)	Encoding Gene	Gene Function
QXBT1	KY994043	ugucuguucccugucucucuag	chr10:48966435-48966526	−38.7	*WDFY4*	exonic
QXBT2	KY994044	ugugucccuaugaaucucaugu	chr2:94772066-94772132	−22.9	*ANKRD20A8P*	ncRNA_intronic
QXBT3	KY994045	uaccucucccaaaacucaugugga	chr9:40297854-40297928	−33.4	*ANKRD20A3*; *FAM95B1*	intergenic
QXBT4	KY994046	uucccucacuguaaacagagu	chr16:21647236-21647297	−24.1	*IGSF6*; *METTL9*	exonic
QXBT5	KY994047	ucugucccaggcccagacuu	chr4:77048486-77048550	−24.7	*CCNI*	exonic
QXBT6	KY994048	aacaggccuugcucugcucacaga	chr3:52523356-52523428	−44.7	*STAB1*	exonic
QXBT7	KY994049	ucucucucucuccgugucagugu	chr10:48827482-48827544	−34.3	*WDFY4*	intronic
QXBT8	KY994050	agggagcggagaagcgagcgcag	chr15:45167265-45167327	−40.2	*SHF*	3′UTR
QXBT9	KY994051	gaagcagcgccugucgcaacucg	chr17:78140754-78140815	−30.6	*TMC8*	intronic
QXBT10	KY994052	uaauguaguugccacuaggaga	chr1:19910424-19910514	−24.5	*OTUD3*	3′UTR
QXBT11	KY994053	aaaagcuguccacuguagaguu	chr9:32456301-32456370	−34.6	*DDX58*	3′UTR
QXBT12	KY994054	cucccacugcuucacuugacuag	chr4:165400665-165400731	−23.6	*CPE*	intronic
QXBT13	KY994055	ccugucugagcgucgcu	chr14:16057563-16057625	−21.7	NA	intergenic
QXBT14	KY994056	acugugguaauucuagagcu	chr22:11629695-11629789	−8.4	NA; *LOC102723769*	intergenic
QXBT15	KY994057	aggacuggaugucgggcugcau	chr4:141707942-141708053	−22.6	*IL15*	intronic
QXBT16	KY994058	uuuugugugucagggugcaggu	chr14:94113640-94113698	−21.9	*IFI27*	intronic
QXBT17	KY994059	gcgggagaggcggguggac	chr2:136117376-136117461	−45.5	*CXCR4*	intronic
QXBT18	KY994060	acuccucauuuguaaacucagg	chrX:129790441-129790501	−36.8	*SASH3*	intronic
QXBT19	KY994061	uccgcugcagccccucgacgu	chr8:55880664-55880722	−33.1	*LYN*	intronic
QXBT20	KY994062	acguggacuccagacucucugu	chr17:42311190-42311260	−30.5	*STAT5A*	3′UTR
QXBT21	KY994063	acccucaguccguauuggucucu	chr17:7306830-7306888	−32.8	*EIF5A*	upstream
QXBT22	KY994064	aaagcccgugggggaccuguuc	chr22:17108635-17108691	−22.2	*IL17RA*	exonic

## Supplementary Materials

Supplementary materials can be found at www.mdpi.com/1422-0067/18/5/925/s1.

## Abbreviations

27DCs	Monocyte-derived IL-27 treated immature dendritic cells
APCs	Antigen presenting cells
DCs	Dendritic cells
EBI-3	Epstein-Barr virus-induced gene 3
FDR	False discovery rate
GO	Gene ontology
iDCs	Monocyte-derived immature DCs
IL-27	Interleukin-27
JAK/STAT	Janus kinase/signal transducer and activator of transcription
JNK	C-Jun N-terminal kinases
KEGG	Kyoto encyclopedia of genes and genomes
logCPM	log2 counts per million
MAPKs	Mitogen-activated protein kinases
MFE	Minimum free energy
miRNAs	MicroRNAs
PI3K	Phosphoinositide 3-kinase
piRNAs	Piwi-interacting RNAs
RT-qPCR	Quantitative reverse transcription polymerase chain reaction
TLR	toll-like receptors

## References

[1] Liu K., Nussenzweig M.C. (2010). Origin and development of dendritic cells. Immunol. Rev..

[2] Banchereau J., Briere F., Caux C., Davoust J., Lebecque S., Liu Y.J., Pulendran B., Palucka K. (2000). Immunobiology of dendritic cells. Ann. Rev. Immunol..

[3] Wu L., KewalRamani V.N. (2006). Dendritic-cell interactions with HIV: Infection and viral dissemination. Nat. Rev. Immunol..

[4] Coleman C.M., Wu L. (2009). HIV interactions with monocytes and dendritic cells: Viral latency and reservoirs. Retrovirology.

[5] Kim V.N. (2005). MicroRNA biogenesis: Coordinated cropping and dicing. Nat. Rev. Mol. Cell Biol..

[6] Bartel D.P. (2009). MicroRNAs: Target recognition and regulatory functions. Cell.

[7] Pritchard C.C., Cheng H.H., Tewari M. (2012). MicroRNA profiling: Approaches and considerations. Nat. Rev. Genet..

[8] Schwarzenbach H., Nishida N., Calin G.A., Pantel K. (2014). Clinical relevance of circulating cell-free microRNAs in cancer. Nat. Rev. Clin. Oncol..

[9] Turner M.L., Schnorfeil F.M., Brocker T. (2011). MicroRNAs regulate dendritic cell differentiation and function. J. Immunol..

[10] Chen C.Z., Schaffert S., Fragoso R., Loh C. (2013). Regulation of immune responses and tolerance: The microRNA perspective. Immunol. Rev..

[11] Montecalvo A., Larregina A.T., Shufesky W.J., Stolz D.B., Sullivan M.L., Karlsson J.M., Baty C.J., Gibson G.A., Erdos G., Morelli A.E. (2012). Mechanism of transfer of functional microRNAs between mouse dendritic cells via exosomes. Blood.

[12] Mittelbrunn M., Gutierrez-Vazquez C., Villarroya-Beltri C., Gonzalez S., Sanchez-Cabo F., Gonzalez M.A., Bernad A., Sanchez-Madrid F. (2011). Unidirectional transfer of microRNA-loaded exosomes from T cells to antigen-presenting cells. Nat. Commun..

[13] Swaminathan G., Navas-Martin S., Martin-Garcia J. (2014). MicroRNAs and HIV-1 infection: Antiviral activities and beyond. J. Mol. Biol..

[14] Stumhofer J.S., Hunter C.A. (2008). Advances in understanding the anti-inflammatory properties of IL-27. Immunol. Lett..

[15] Pflanz S., Timans J.C., Cheung J., Rosales R., Kanzler H., Gilbert J., Hibbert L., Churakova T., Travis M., Kastelein R.A. (2002). IL-27, a heterodimeric cytokine composed of EBI3 and p28 protein, induces proliferation of naive CD4+ T cells. Immunity.

[16] Lucas S., Ghilardi N., Li J., de Sauvage F.J. (2003). IL-27 regulates IL-12 responsiveness of naive CD4+ T cells through Stat1-dependent and -independent mechanisms. Proc. Natl. Acad. Sci. USA.

[17] Hibbert L., Pflanz S., de Waal Malefyt R., Kastelein R.A. (2003). IL-27 and IFN-α signal via Stat1 and Stat3 and induce T-Bet and IL-12Rβα2 in naive T cells. J. Interferon Cytokine Res..

[18] Vignali D.A., Kuchroo V.K. (2012). IL-12 family cytokines: Immunological playmakers. Nat. Immunol..

[19] Chen Q., Swaminathan S., Yang D., Dai L., Sui H., Yang J., Hornung R.L., Wang Y., Hu X., Imamichi T. (2013). Interleukin-27 is a potent inhibitor of cis HIV-1 replication in monocyte-derived dendritic cells via a type I interferon-independent pathway. PLoS ONE.

[20] Dai L., Lidie K.B., Chen Q., Adelsberger J.W., Zheng X., Huang D., Yang J., Lempicki R.A., Lane H.C., Imamichi T. (2013). IL-27 inhibits HIV-1 infection in human macrophages by down-regulating host factor SPTBN1 during monocyte to macrophage differentiation. J. Exp. Med..

[21] Jia H., Dilger P., Bird C., Wadhwa M. (2016). IL-27 Promotes proliferation of human leukemic cell lines through the MAPK/ERK signaling pathway and suppresses sensitivity to chemotherapeutic drugs. J. Interferon Cytokine Res..

[22] Diegelmann J., Olszak T., Goke B., Blumberg R.S., Brand S. (2012). A novel role for interleukin-27 (IL-27) as mediator of intestinal epithelial barrier protection mediated via differential signal transducer and activator of transcription (STAT) protein signaling and induction of antibacterial and anti-inflammatory proteins. J. Biol. Chem..

[23] Sharma G., Dutta R.K., Khan M.A., Ishaq M., Sharma K., Malhotra H., Majumdar S. (2014). IL-27 inhibits IFN-γ induced autophagy by concomitant induction of JAK/PI3 K/Akt/mTOR cascade and up-regulation of Mcl-1 in Mycobacterium tuberculosis H37Rv infected macrophages. Int. J. Biochem. Cell Biol..

[24] Fakruddin J.M., Lempicki R.A., Gorelick R.J., Yang J., Adelsberger J.W., Garcia-Pineres A.J., Pinto L.A., Lane H.C., Imamichi T. (2007). Noninfectious papilloma virus-like particles inhibit HIV-1 replication: Implications for immune control of HIV-1 infection by IL-27. Blood.

[25] Sowrirajan B., Saito Y., Poudyal D., Chen Q., Sui H., DeRavin S.S., Imamichi H., Malech H.L., Lane H.L., Imamichi T. (2017). Interleukin-27 Enhances the potential of reactive oxygen species generation from monocyte-derived macrophages and dendritic cells by induction of p47phox. Sci. Rep..

[26] Love M.I., Huber W., Anders S. (2014). Moderated estimation of fold change and dispersion for RNA-seq data with DESeq2. Genome Biol..

[27] Vlachos I.S., Kostoulas N., Vergoulis T., Georgakilas G., Reczko M., Maragkakis M., Paraskevopoulou M.D., Prionidis K., Dalamagas T., Hatzigeorgiou A.G. (2012). DIANA miRPath v.2.0: Investigating the combinatorial effect of microRNAs in pathways. Nucleic Acids Res..

[28] Agarwal V., Bell G.W., Nam J.W., Bartel D.P. (2015). Predicting effective microRNA target sites in mammalian mRNAs. eLife.

[29] Abdalla A.E., Li Q., Xie L., Xie J. (2015). Biology of IL-27 and its role in the host immunity against Mycobacterium tuberculosis. Int. J. Biol. Sci..

[30] Kamiya S., Owaki T., Morishima N., Fukai F., Mizuguchi J., Yoshimoto T. (2004). An indispensable role for STAT1 in IL-27-induced T-bet expression but not proliferation of naive CD4+ T cells. J. Immunol..

[31] Takeda A., Hamano S., Yamanaka A., Hanada T., Ishibashi T., Mak T.W., Yoshimura A., Yoshida H. (2003). Cutting edge: Role of IL-27/WSX-1 signaling for induction of T-bet through activation of STAT1 during initial Th1 commitment. J. Immunol..

[32] Blahoianu M.A., Rahimi A.A., Kozlowski M., Angel J.B., Kumar A. (2014). IFN-γ-induced IL-27 and IL-27p28 expression are differentially regulated through JNK MAPK and PI3K pathways independent of Jak/STAT in human monocytic cells. Immunobiology.

[33] Heikkilä O., Nygårdas M., Paavilainen H., Ryödi E., Hukkanen V. (2016). Interleukin-27 inhibits herpes simplex virus type 1 infection by activating STAT1 and 3, interleukin-6, and chemokines IP-10 and MIG. J. Interferon Cytokine Res..

[34] Swaminathan S., Hu X., Zheng X., Kriga Y., Shetty J., Zhao Y., Stephens R., Tran B., Lane H.C., Imamichi T. (2013). Interleukin-27 treated human macrophages induce the expression of novel microRNAs which may mediate anti-viral properties. Biochem. Biophys. Res. Commun..

[35] Lecellier C.H., Dunoyer P., Arar K., Lehmann-Che J., Eyquem S., Himber C., Saib A., Voinnet O. (2005). A cellular microRNA mediates antiviral defense in human cells. Science.

[36] John B., Enright A.J., Aravin A., Tuschl T., Sander C., Marks D.S. (2004). Human microRNA targets. PLoS Biol..

[37] Friedländer M.R., Mackowiak S.D., Li N., Chen W., Rajewsky N. (2012). miRDeep2 accurately identifies known and hundreds of novel microRNA genes in seven animal clades. Nucleic Acids Res..

[38] Park H., Huang X., Lu C., Cairo M.S., Zhou X. (2015). MicroRNA-146a and microRNA-146b regulate human dendritic cell apoptosis and cytokine production by targeting TRAF6 and IRAK1 proteins. J. Biol. Chem..

[39] Zhang M., Liu F., Jia H., Zhang Q., Yin L., Liu W., Li H., Yu B., Wu J. (2011). Inhibition of microRNA let-7i depresses maturation and functional state of dendritic cells in response to lipopolysaccharide stimulation via targeting suppressor of cytokine signaling 1. J. Immunol..

[40] Krichevsky A.M., Gabriely G. (2009). miR-21: A small multi-faceted RNA. J. Cell. Mol. Med..

[41] Sheedy F.J. (2015). Turning 21: Induction of miR-21 as a Key Switch in the Inflammatory Response. Front. Immunol..

[42] Smyth L.A., Boardman D.A., Tung S.L., Lechler R., Lombardi G. (2015). MicroRNAs affect dendritic cell function and phenotype. Immunology.

[43] Hashimi S.T., Fulcher J.A., Chang M.H., Gov L., Wang S., Lee B. (2009). MicroRNA profiling identifies miR-34a and miR-21 and their target genes JAG1 and WNT1 in the coordinate regulation of dendritic cell differentiation. Blood.

[44] Lu C., Huang X., Zhang X., Roensch K., Cao Q., Nakayama K.I., Blazar B.R., Zeng Y., Zhou X. (2011). miR-221 and miR-155 regulate human dendritic cell development, apoptosis, and IL-12 production through targeting of p27kip1, KPC1, and SOCS-1. Blood.

[45] Woltman A.M., van der Kooij S.W., Coffer P.J., Offringa R., Daha M.R., van Kooten C. (2003). Rapamycin specifically interferes with GM-CSF signaling in human dendritic cells, leading to apoptosis via increased p27KIP1 expression. Blood.

[46] Mayoral R.J., Pipkin M.E., Pachkov M., van Nimwegen E., Rao A., Monticelli S. (2009). MicroRNA-221–222 regulate the cell cycle in mast cells. J. Immunol..

[47] Kuipers H., Schnorfeil F.M., Brocker T. (2010). Differentially expressed microRNAs regulate plasmacytoid vs. conventional dendritic cell development. Mol. Immunol..

[48] Pflanz S., Hibbert L., Mattson J., Rosales R., Vaisberg E., Bazan J.F., Phillips J.H., McClanahan T.K., de Waal Malefyt R., Kastelein R.A. (2004). WSX-1 and glycoprotein 130 constitute a signal-transducing receptor for IL-27. J. Immunol..

[49] Liu L., Cao Z., Chen J., Li R., Cao Y., Zhu C., Wu K., Wu J., Liu F., Zhu Y. (2012). Influenza A virus induces interleukin-27 through cyclooxygenase-2 and protein kinase A signaling. J. Biol. Chem..

[50] Frank A.C., Zhang X., Katsounas A., Bharucha J.P., Kottilil S., Imamichi T. (2010). Interleukin-27, an anti-HIV-1 cytokine, inhibits replication of hepatitis C virus. J. Interferon Cytokine Res..

[51] Ten Oever B.R. (2013). RNA viruses and the host microRNA machinery. Nat. Rev. Microbiol..

[52] Martin M. (2011). Cutadapt removes adapter sequences from high-throughput sequencing reads. EMBnet. J..

[53] Li H., Durbin R. (2009). Fast and accurate short read alignment with Burrows-Wheeler transform. Bioinformatics.

[54] Tam S., Tsao M.S., McPherson J.D. (2015). Optimization of miRNA-seq data preprocessing. Brief. Bioinform..

[55] Quinlan A.R., Hall I.M. (2010). BEDTools: A flexible suite of utilities for comparing genomic features. Bioinformatics.

[56] Griffiths-Jones S., Grocock R.J., van Dongen S., Bateman A., Enright A.J. (2006). miRBase: MicroRNA sequences, targets and gene nomenclature. Nucleic Acids Res..

[57] Robinson M.D., McCarthy D.J., Smyth G.K. (2010). edgeR: A bioconductor package for differential expression analysis of digital gene expression data. Bioinformatics.

[58] Kolde R. Pheatmap: Pretty Heatmaps, R Package Version 1.0.8, 2015. https://cran.r-project.org/web/packages/pheatmap/index.html.

[59] Kanehisa M., Goto S., Sato Y., Furumichi M., Tanabe M. (2012). KEGG for integration and interpretation of large-scale molecular data sets. Nucleic Acids Res..

[60] Vlachos I.S., Zagganas K., Paraskevopoulou M.D., Georgakilas G., Karagkouni D., Vergoulis T., Dalamagas T., Hatzigeorgiou A.G. (2015). DIANA-miRPath v3.0: Deciphering microRNA function with experimental support. Nucleic Acids Res..

[61] Wang K., Li M., Hakonarson H. (2010). ANNOVAR: Functional annotation of genetic variants from high-throughput sequencing data. Nucleic Acids Res..

[62] Reiche K., Stadler P.F. (2007). RNAstrand: Reading direction of structured RNAs in multiple sequence alignments. Algorithms Mol. Biol. AMB.

[63] Livak K.J., Schmittgen T.D. (2001). Analysis of relative gene expression data using real-time quantitative PCR and the 2^−ΔΔ*C*t^ Method. Methods.

