# Supplementary material for: Genome-Wide Analyses of MicroRNA Profiling in Interleukin-27 Treated Monocyte-Derived Human Dendritic Cells Using Deep Sequencing: A Pilot Study
Source: Int J Mol Sci. 2017 Apr 28;18(5):925. doi: 10.3390/ijms18050925 (PMC5454838; doi:10.3390/ijms18050925)
Supplement: Supplementary file 1 [file ijms-18-00925-s001.zip › Supplementaly materials 1.pdf]

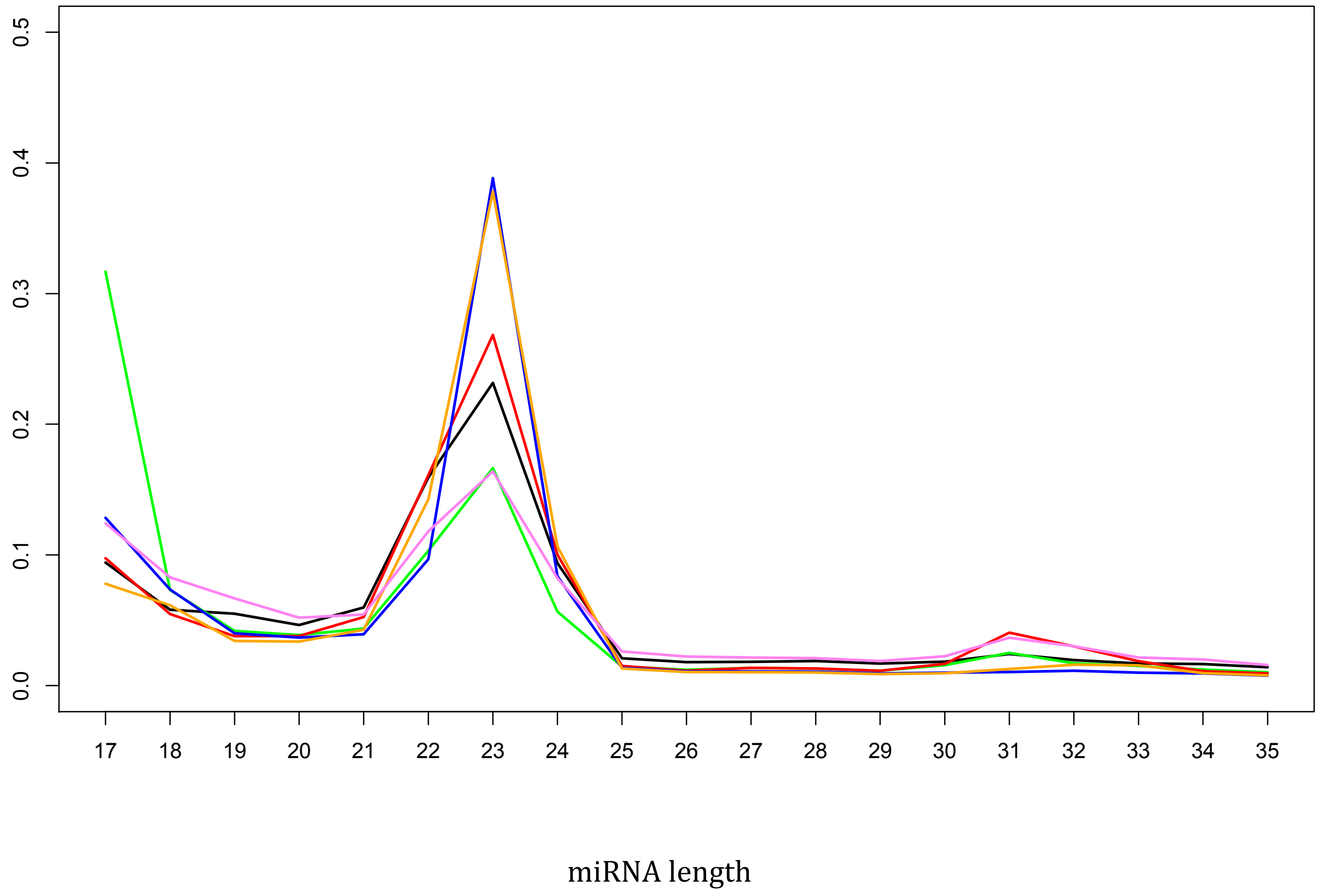

Figure S1. Length distribution of microRNAs (miRNAs) reads from immature dendritic cells (iDCs) and IL-27-treated iDCs (27DCs). Different colors represent different samples.

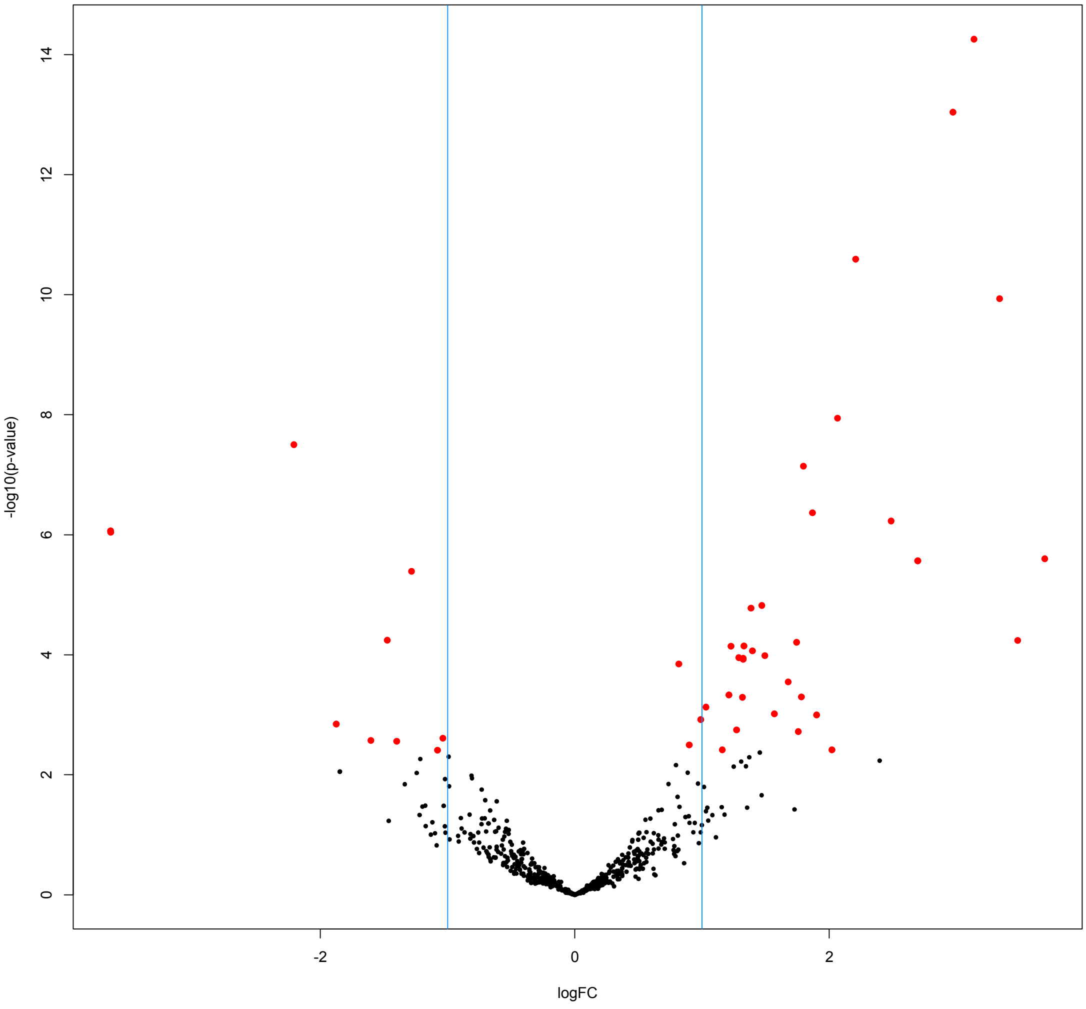

Figure S2. Volcano plot of known MicroRNAs (miRNAs) profiles. The x-axis shows the log2fold-change of IL-27-treated iDCs (27DCs) vs. immature dendritic cells (iDCs), while the y-axis shows the –log10 P-value of miRNA. The red point in the plot represents the differentially expressed miRNAs with statistical significance. The vertical lines correspond to a two-fold change in expression (up or down).

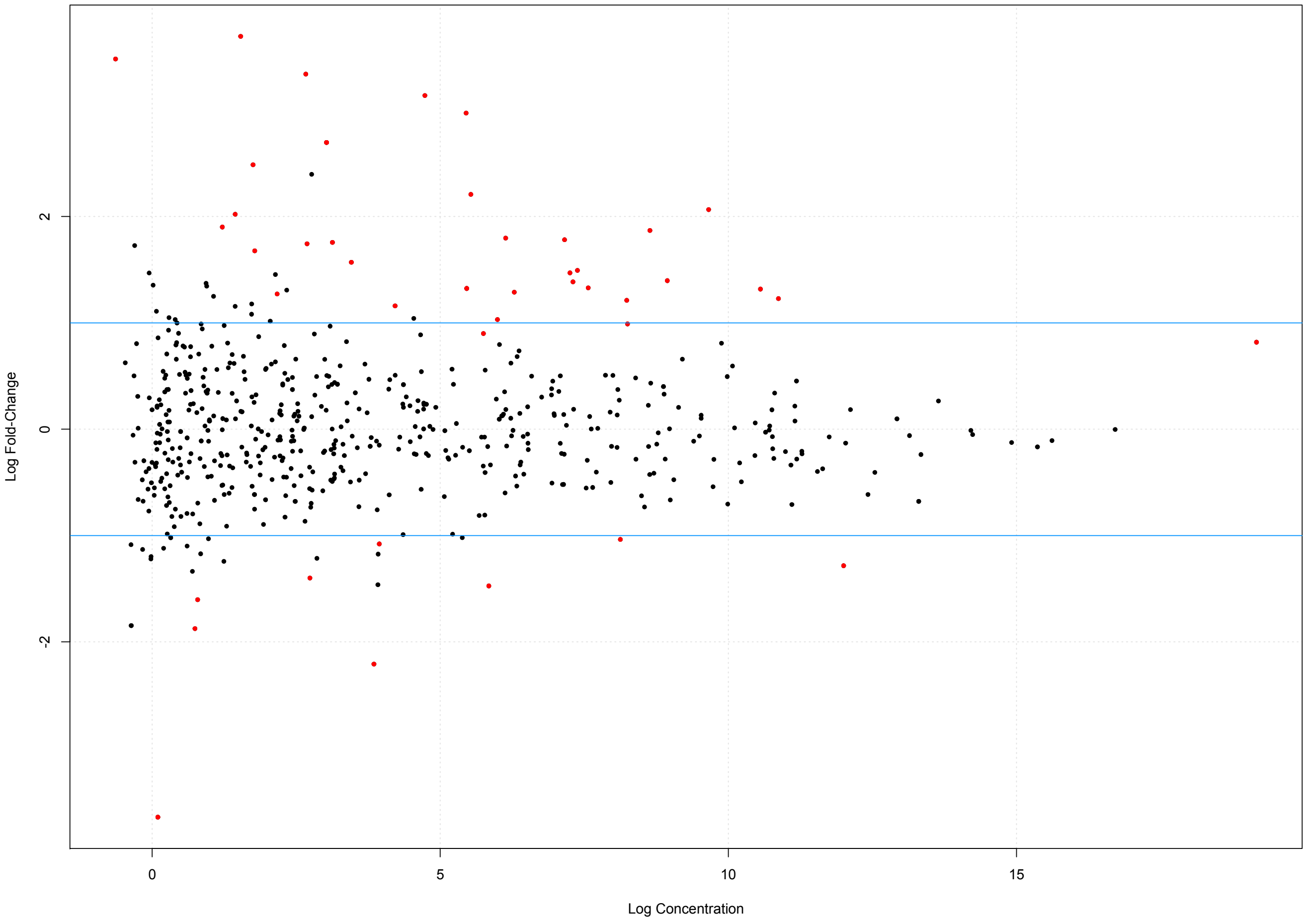

Figure S3. M-A plot. The MA plot shows for each microRNA (miRNA) (dot) the difference in expression levels between immature dendritic cells (iDCs) and IL-27-treated iDCs (27DCs) (M, log2 fold change; y axis) compared to their mean expression level (A, log2 transformed normalized miRNA read count; x axis). Blue line indicates twofold difference in expression.

A.

Provisional ID	:	chr10_2389
Score total	:	195.8
Score for star read(s)	:	3.9
Score for read counts	:	189.3
Score for mfe	:	1.7
Score for randfold	:	1.6
Score for cons. seed	:	-0.6
Total read count	:	383
Mature read count	:	358
Loop read count	:	0
Star read count	:	25

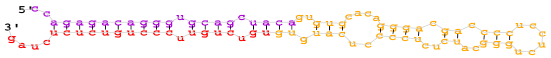

QXBT1

chr10:48966435..48966526:+

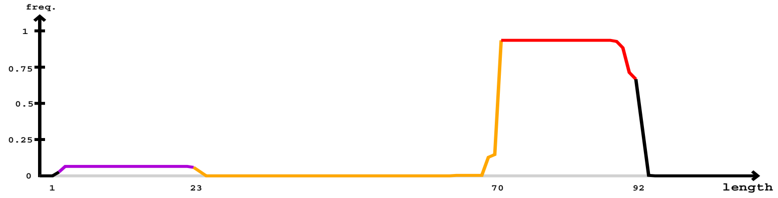


```
                          Star                                                       Mature
5'- cccagagacagggugcagcuacagugugcacagggacgaccccucccucugggcaucucuccccucaugugugucuguucccugucucucuagcucuuuaccaaaccucac  -3'   obs
    cccagagacagggugcagcuacagugugcacagggacgaccccucccucugggcaucucuccccucaugugugucuguucccugucucucuagcucuuuaccaaaccucac         exp
    ...(((((((((.((((.((((..(((....((((.((.(((......)))..))..))))..))).)))).)))).))))))))).........................   reads   mm   sample
    .ccagagacagggugcagcuac.........................................................................................   1       0    iD7
    .Acagagacagggugcagcuaca........................................................................................   1       1    iD7
    .ccagagacagggugcagcuaca........................................................................................   6       0    iD7
    .ccagagacagggugcagcuacU........................................................................................   2       1    iD7
    ..cagagacaggAugcagcuac.........................................................................................   1       1    iD7
    ..cagagacagggugcagcuac.........................................................................................   1       0    iD7
    ..cagagacagAgugcagcuaca........................................................................................   1       1    iD7
    ..cagagacagggugcagcuaca........................................................................................   4       0    iD7
    ..cagagacagggugcagcuacG........................................................................................   1       1    iD7
    ..cagagacagggugcagcuacaU.......................................................................................   1       1    iD7
    ..cagagacagggugcagcuacag.......................................................................................   5       0    iD7
    ..cagagacagggugcagcuacaA.......................................................................................   1       1    iD7
    ................................................................ucaugugugucuguucccugucucuc.....................   1       0    iD7
    .....................................................................ugugucuguucccugucucu......................   2       0    iD7
    .....................................................................ugugucuguucccugucucA......................   1       1    iD7
    .....................................................................ugugCcuguucccugucucuc.....................   1       1    iD7
    .....................................................................ugugucuguucccugucucuc.....................   10      0    iD7
    .....................................................................ugugucuguucccugucucuU.....................   1       1    iD7
    .....................................................................ugugucuguucccugucucuUu....................   1       1    iD7
    .....................................................................ugugucuguucccugucucucC....................   6       1    iD7
    .....................................................................ugugucuguucccugucucucG....................   2       1    iD7
    .....................................................................ugugucuUuucccugucucucu....................   1       1    iD7
    .....................................................................ugugucuguucccugucucucu....................   12      0    iD7
    .....................................................................ugugucuguucccugucucucA....................   5       1    iD7
    .....................................................................ugugucuguucccugucucucua...................   1       0    iD7
    .....................................................................ugugucuguucccugucucucAa...................   2       1    iD7
    .....................................................................ugugucuguucccugucucucuag..................   1       0    iD7
    .....................................................................ugugucuguucccugucucucuaU..................   2       1    iD7
    ......................................................................gugucuguucccugucucuA.....................   1       1    iD7
    ......................................................................gugucuguucccugucucucu....................   3       0    iD7
    ......................................................................gugucuguucccugucucucuU...................   1       1    iD7
    ......................................................................Augucuguucccugucucucua...................   1       1    iD7
    ......................................................................gugucuguucccugucucucuaC..................   1       1    iD7
```

B.

Provisional ID	: chr2_7214
Score total	: 1.1
Score for star read(s)	: -1.3
Score for read counts	: -2.3
Score for mfe	: 0.2
Score for randfold	: 1.6
Score for cons. seed	: 3
Total read count	: 7
Mature read count	: 7
Loop read count	: 0
Star read count	: 0

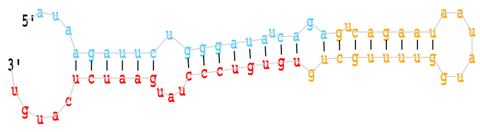

QXBT2

chr2:94772066..94772132:-

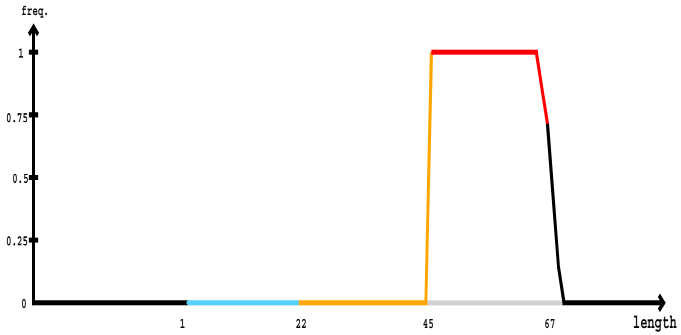

Star Mature

```
5'- auugcauuuugcucugugauaaggacauaagauucugggauaucagagucagaauaauaugguuuugcugugugucccuaugaaucucauguggaauuguaaucccuaaugu  -3'   exp
    ((((((........))))))....((((.((((((.((((((.((.((.((((((......)))))))).))))))))...)))))).))))(((.......)))......   reads   mm   sample
    ......................................................................ugugucccuaugaaucucau......................   1       0    iD4
    ......................................................................ugugucccuaugaaucucaug.....................   1       0    iD4
    ......................................................................ugugucccuaugaaucucaugu....................   4       0    iD4
    ......................................................................ugugucccuaugaaucucaugugU..................   1       1    iD4
```

c.

Provisional ID	:	chr9_19385
Score total	:	1.4
Score for star read(s)	:	-1.3
Score for read counts	:	0
Score for mfe	:	1.7
Score for randfold	:	1.6
Score for cons. seed	:	-0.6
Total read count	:	155
Mature read count	:	92
Loop read count	:	10
Star read count	:	53

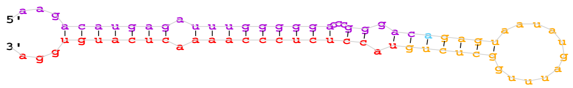

QXBT3

chr9:40297854..40297928:+

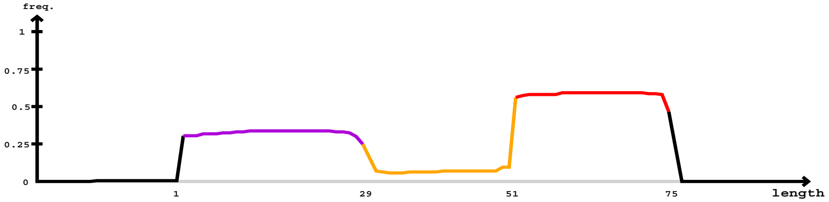

Star Mature

```
5'- uuguauuuugcaaugugagaaagacaugagauuugggggacccgggacagaguaauaugauuuggcucuguaccucucccaaaacucaugugga auuuuaauggggaauguuaa -3'   obs
    uuguauuuugcaaugugagaaagacaugagauuugggggacccgggacagaguaauaugauuuggcucuguaccucucccaaaacucauguggaauuuuaauggggaauguuaa        exp
    ...((((((.((...((((...(((((((.((((((((...((.((((((..........))))))).))))))))))).)))))))....))))).)).))))))....   reads   mm   sample
    .......uugcaaugugagaaGgaca........................................................................................   1   1   iD7
    ....................aagacaugagauuugggggaccc.......................................................................   1   0   iD7
    ....................aagacaugagauuugggggacccgg......................................................................   1   0   iD7
    ....................aagacaugagauuugggggacccggg.....................................................................   4   0   iD7
    ....................aagacaugagauuugggggacccgggU....................................................................   1   1   iD7
    ....................aagacaugagauuugggggacccggga....................................................................   7   0   iD7
    ....................aagacaugagauuugggggacccgggaA...................................................................   1   1   iD7
    ....................aagacaugagauuugggggacccgggac...................................................................   18  0   iD7
    ....................aagacaugagauuugggggacccgggaU...................................................................   3   1   iD7
    ....................aagacaugagauuugggggaccAgggaca.................................................................   1   1   iD7
    ....................aagacaugagauuugggggacccgggaca.................................................................   7   0   iD7
    ....................aagacaugagauuugggggacccgggaUa.................................................................   1   1   iD7
    ....................aagacaugagauuugggggacccgggacC..................................................................   1   1   iD7
    ....................aagacaugagauuugggggacccgggacU..................................................................   1   1   iD7
    .......................acaugagauuugggggaccAgggaca.................................................................   2   1   iD7
    ..........................ugagauuugggggaccAgggaca.................................................................   1   1   iD7
    ..........................ugagauuugggggacccgggaca.................................................................   1   0   iD7
    ............................agauuugggggaccAgggacaga...............................................................   1   1   iD7
    ..............................auuugggggaccAgggacag................................................................   1   1   iD7
    .................................................gaguaauaugauuuggcucug............................................   8   0   iD7
    .................................................gaguaauaugauuuggcucuA............................................   1   1   iD7
    ......................................................auaugauuuggcuGugu...........................................   1   1   iD7
    ...........................................................auuuggcucuguaccucucccaaaacucaugugga....................   1   0   iD7
    ....................................................................uguaccucucccaaaacucaug........................   1   0   iD7
    ....................................................................uguaccucucccaaaacucauguggG....................   1   1   iD7
    ....................................................................uguaccucucccaaaacucaugugga....................   2   0   iD7
    ......................................................................uaccucucccaaaacucaugug......................   1   0   iD7
    ......................................................................uaccucucccaaaacucaugugg.....................   13  0   iD7
    ......................................................................uaccucucccaaaacucaugugC.....................   1   1   iD7
    ......................................................................uaccCcucccaaaacucaugugg.....................   1   1   iD7
    ......................................................................uaccucucccaaaacucaugugA.....................   3   1   iD7
    ......................................................................uUccucucccaaaacucaugugga....................   1   1   iD7
    ......................................................................uaccucucccCaaacucaugugga....................   1   1   iD7
```

D.

Provisional ID	: chr16_4548
Score total	: 0.9
Score for star read(s)	: -1.3
Score for read counts	: 0
Score for mfe	: 1.2
Score for randfold	: 1.6
Score for cons. seed	: -0.6
Total read count	: 39
Mature read count	: 39
Loop read count	: 0
Star read count	: 0

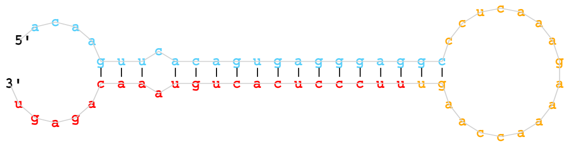

QXBT4

chr16:21647236..21647297:-

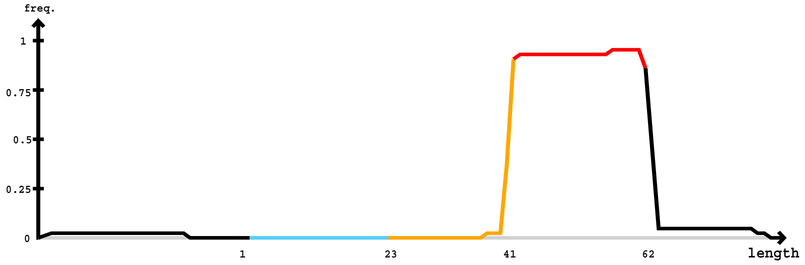

Star Mature

```
5'- ugugcuuggacgggugcaaaagugaggcagacaaguucacagugagggaggcccucaaagaaaaccaaguuucccucacuguaaacagagugacuucaaaugacagugcaa  -3'   exp
    (((((((....)))))))....((((((..(..(((.((((((((((((((...............)))))))))))))).))).)..)).))))).............      reads   mm      sample
    ugugcuuggacgggugcaaaa..........................................................................................      1       0       DC4
    ..................................................................aaguuucccucacuguaaaca........................      1       0       DC4
    .....................................................................uuucccucacuguaaacagag.....................      1       0       DC4
    .....................................................................uuucccucacuguaaacagaA.....................      1       1       DC4
    .....................................................................uuucccucacuguaaacagagC....................      3       1       DC4
    .....................................................................uuucccucacuguaaacagagu....................      10      0       DC4
    ......................................................................uucccucacuguaaacagag.....................      2       0       DC4
    ......................................................................uucccucacuguaaacagaAu....................      1       1       DC4
    ......................................................................uucccucacuguaaacagagu....................      15      0       DC4
    ......................................................................uucccucacuguaaacagagC....................      4       1       DC4
    ......................................................................uucccucacuguaaacagaguU...................      1       1       DC4
    .......................................................................ucccucacuguaaacagagu....................      1       0       DC4
    .....................................................................................cagagugacuucaaaugacagu....      1       0       DC4
    .......................................................................................gagugacuucaaaugacagugc..      1       0       DC4
```

E.

Provisional ID	:	chr4_10978
Score total	:	65.1
Score for star read(s)	:	3.9
Score for read counts	:	56.7
Score for mfe	:	0
Score for randfold	:	1.6
Score for cons. seed	:	3
Total read count	:	123
Mature read count	:	63
Loop read count	:	0
Star read count	:	60

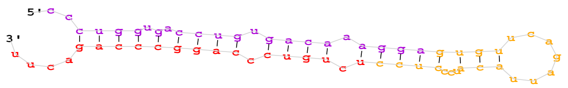

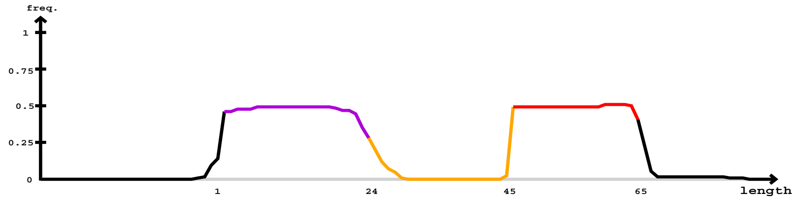

QXBT5

chr4:77048486..77048550:-

```
                              Star                                        Mature
5'- uuaugucuaccguccccucaagcacacccuggugaccugugacaaaggaguguucagauuacaucccuccucugucccaggcccagacuucuccaaggacaacagcaagc  -3'  obs
    uuaugucuaccguccccucaagcacacccuggugaccugugacaaaggaguguucagauuacaucccuccucugucccaggcccagacuucuccaaggacaacagcaagc       exp
    ...((.((...((((.............((((.(.((((.((((.((((((((.......)))...))))).)))).)))))))))..........))))...))))...       reads  mm  sample
    ......................cacacccuggugaccugugac...................................................................       1      0   iD2
    .......................acacccuggugaccugugaca..................................................................       1      0   iD2
    ........................cacccuggugaccugugacaaa................................................................       2      0   iD2
    ........................cacccuggugaccugugacaaag...............................................................       7      0   iD2
    ........................cacccuggugaccugugacaaagA..............................................................       1      1   iD2
    .........................acccuggugaccugugaca..................................................................       1      0   iD2
    .........................acccuggugaccugugacaaag...............................................................       2      0   iD2
    .........................acccuggugaccugugacaaagU..............................................................       1      1   iD2
    .........................acccuggugaccugugacaaagga.............................................................       1      0   iD2
    .........................acccuggugaccugugacaaaggagu...........................................................       1      0   iD2
    ..........................cccuggugaccugugacaaa................................................................       1      0   iD2
    ..........................cccuggugaccugugacaaag...............................................................       3      0   iD2
    ..........................cccuggugaccugugacaaagg..............................................................       7      0   iD2
    ..........................Uccuggugaccugugacaaagga.............................................................       1      1   iD2
    ..........................cccuggugaccugugacaaaggU.............................................................       1      1   iD2
    ..........................cccuggugaccugugacaaagga.............................................................       13     0   iD2
    ..........................cccuggugaccugugacaaaggG.............................................................       2      1   iD2
    ..........................cccuggugaccugugacaaaggag............................................................       2      0   iD2
    ..........................cccuggugaccugugacaaaggaA............................................................       1      1   iD2
    ..........................cccuggugaccugugacaaaggagu...........................................................       2      0   iD2
    ..........................cccuggugaccugugacaaaggaUu...........................................................       1      1   iD2
    ..........................cccuggugaccugugacaaaggagug..........................................................       1      0   iD2
    ..........................cccuggugaccugugacaaaggagugu.........................................................       4      0   iD2
    ..........................cccuggugaccugugacaaaggagugC.........................................................       1      1   iD2
    ..........................cccuggugaccugugacaaaggaguguC........................................................       1      1   iD2
    ............................cuggugaccugugacaaaggGgu...........................................................       1      1   iD2
    ............................cuggugaccugugacaaaggagu...........................................................       1      0   iD2
    ...............................gugaccugugacaaaggagug..........................................................       2      0   iD2
    .....................................................................cucugucccaggcccagacuC....................       1      1   iD2
    .....................................................................cucugucccaggcccagacuu....................       2      0   iD2
    ......................................................................ucugucccaggcccagac......................       1      0   iD2
    ......................................................................ucugucccaggcccagacu.....................       11     0   iD2
    ......................................................................ucugucccaggcccagacC.....................       1      1   iD2
```

F.

Provisional ID	: chr3_8327
Score total	: 1.9
Score for star read(s)	: -1.3
Score for read counts	: 0
Score for mfe	: 2.2
Score for randfold	: 1.6
Score for cons. seed	: -0.6
Total read count	: 19
Mature read count	: 19
Loop read count	: 0
Star read count	: 0

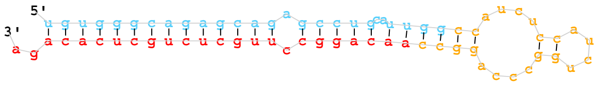

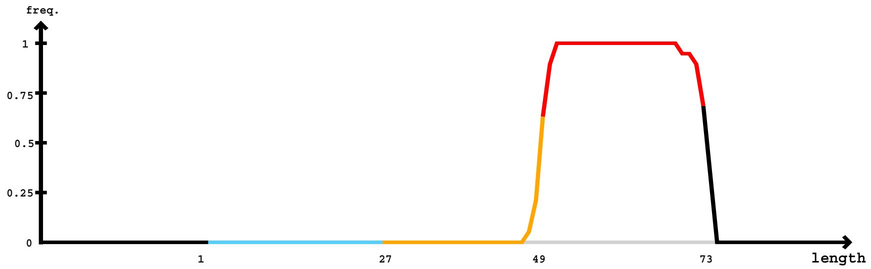

QXBT6

chr3:52523356..52523428:+

```
                    Star                                        Mature
5'- acaacaugguaacccccaagggugugggcagagcagagccugcauuggccaucuccaucuggcccaggccaacaggccuugcucugcucacagacgcugaguggcccagacuug  -3'   exp
    ......(((...(((.((.(..(((((((((((((.(((((..((((((....((....))....))))))))))).)))))))))))))..)..)).).)).)))......       reads   mm   sample
    ....................................................................ccaacaggccuugcucugcucU........................       1       1    DC4
    .....................................................................caacaggccuugcucugcucacag.....................       1       0    DC4
    .....................................................................caacaggccuugcucugcucacagU....................       1       1    DC4
    .....................................................................caacaggccuugcucugcucacaga....................       1       0    DC4
    ......................................................................aacaggccuugcucugcucaca......................       1       0    DC4
    ......................................................................aacaggccuugcucugcucacag.....................       1       0    DC4
    ......................................................................aacaggccuugcucugcucacagU....................       2       1    DC4
    ......................................................................aacaggccuugcucugcucacaga....................       3       0    DC4
    ......................................................................aacaggccuugcucugcucacagUc...................       1       1    DC4
    .......................................................................acaggccuugcucugcucacag.....................       2       0    DC4
    .......................................................................acaggccuugcucugcucacagC....................       1       1    DC4
    .......................................................................acaggccuugcucugcucacagU....................       2       1    DC4
    ........................................................................caggccuugcucugcucacagG....................       1       1    DC4
    ........................................................................caggccuugcucugcucacaga....................       1       0    DC4
```

G.

Provisional ID	:	chr10_1977
Score total	:	6.1
Score for star read(s)	:	-1.3
Score for read counts	:	0
Score for mfe	:	2.8
Score for randfold	:	1.6
Score for cons. seed	:	3
Total read count	:	91
Mature read count	:	80
Loop read count	:	0
Star read count	:	11

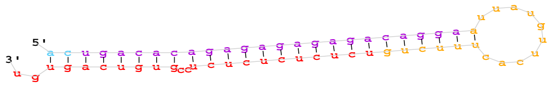

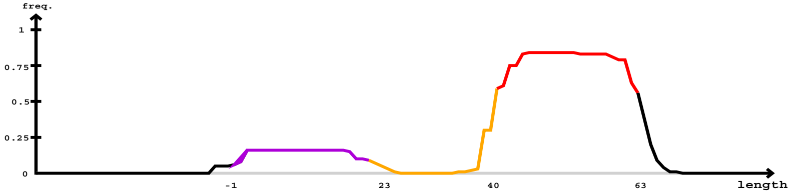

QXBT7

chr10:48827482..48827544:+

```
                          Star                                    Mature
5'- auuuccaaaucugaaugugguucaugcacacugacacagagagagagacaggaauuauguucacuuucugucucucucucuccgugucagugugcaugaaccacauucuccag   -3'   obs
    auuuccaaaucugaaugugguucaugcacacugacacagagagagagacaggaauuauguucacuuucugucucucucucuccgugucagugugcaugaaccacauucuccag         exp
    ............((((((((((((((((((((((((((((((((((((((((((.........))))))))))))))))..))))))))))))))))))))))))))).....     reads   mm   sample
    ..........................cacacugacacagagagagaga.................................................................     5       0    DC7
    .............................acugacacagagagagagacagg.............................................................     1       0    DC7
    ..............................cugacacagagagagag..................................................................     1       0    DC7
    ..............................cugacacagagagagagacU...............................................................     1       1    DC7
    ...............................ugacacagagagagagacag..............................................................     1       0    DC7
    ...............................ugacacagagagagagacagga............................................................     4       0    DC7
    ...............................ugacacagagagagagacaggaa...........................................................     2       0    DC7
    ...............................ugacacagagagagagacaggaau..........................................................     1       0    DC7
    ................................................................uuGcugucucucucucucc..............................     1       1    DC7
    ..................................................................ucugucucucucucuccguguca........................     1       0    DC7
    ...................................................................cugucucucucucuccgugucagu......................     1       0    DC7
    ....................................................................ugucucucucucuccguguc.........................     1       0    DC7
    ....................................................................ugucucucucucuccgugucagG......................     1       1    DC7
    ....................................................................ugucucucCcucuccgugucagu......................     1       1    DC7
    ....................................................................ugucucucucucuccgugucagu......................     6       0    DC7
    ....................................................................ugucucucucucuccgugucagC......................     3       1    DC7
    ....................................................................ugucucCcucucuccgugucagug.....................     1       1    DC7
    ....................................................................ugucucucucucuccgugucagug.....................     4       0    DC7
    ....................................................................ugucucucucucuccgugucaguA.....................     1       1    DC7
    ....................................................................ugucucucucucuccgugucagugu....................     7       0    DC7
    ....................................................................ugucCcucucucuccgugucagugu....................     1       1    DC7
    ....................................................................ugucucucucucuccgugucagugC....................     1       1    DC7
    ......................................................................ucucucucucuccguguc.........................     1       0    DC7
    ......................................................................ucucucucucuccguguca........................     1       0    DC7
    ......................................................................ucucucucucuccgugucagG......................     1       1    DC7
    ......................................................................ucucucucucuccgugucagC......................     1       1    DC7
    ......................................................................ucucucucucuccgugucagug.....................     1       0    DC7
    ......................................................................ucucCcucucuccgugucagugu....................     1       1    DC7
    ......................................................................ucucucucucuccgugucagugu....................     15      0    DC7
    ......................................................................ucucucucucuccgugucagugC....................     4       1    DC7
    ......................................................................ucucucucucuccgugucagugA....................     1       1    DC7
    ......................................................................ucucucucucuccgugucaguguC...................     1       1    DC7
    ......................................................................ucucucucucuccgugucagugug...................     1       0    DC7
```

H.

Provisional ID	: chr15_4958
Score total	: 1.6
Score for star read(s)	: -1.3
Score for read counts	: 0
Score for mfe	: 2.1
Score for randfold	: -2.2
Score for cons. seed	: 3
Total read count	: 22
Mature read count	: 22
Loop read count	: 0
Star read count	: 0

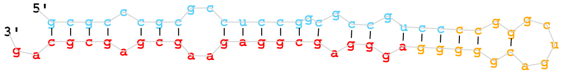

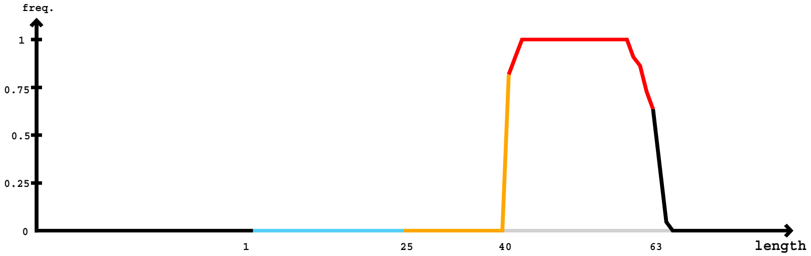

QXBT8

chr15:45167265..45167327:-

```
                                  Star                   Mature
5'- auaaauaaacccgauccugccgggcacagccgcgcccgcgccuccggcgccguccccgggcugacgggggagggagcggagaagcgagcgcagauucugcguauaaaucagcu -3'   exp
    .........((((.......))))(.((((.((((.(((..(((((.(.((.(((((.(.....).))))).)).))))))..))).)))).)...))).)............   reads   mm   sample
    ......................................................................agggagcggagaagcgagc........................   1   0   iD2
    ......................................................................agggagcggagaagcgagA........................   1   1   iD2
    ......................................................................agggagcggagaagcgagcg.......................   1   0   iD2
    ......................................................................agggagcggagaagcgagcgc......................   1   0   iD2
    ......................................................................agggagcggagaagcgagcgU......................   1   1   iD2
    ......................................................................agggagcggagaagcgagcgca.....................   2   0   iD2
    ......................................................................agggagcggagaagcgagcgcag....................   4   0   iD2
    ......................................................................agggagcggagaagcgagcgcaU....................   2   1   iD2
    ......................................................................agggagcggagaagcgagcgcagC...................   1   1   iD2
    ......................................................................agggagcggagaagcgagcgcagU...................   2   1   iD2
    ......................................................................agggagcggagaagcgagcgcaga...................   1   0   iD2
    ......................................................................agggagcggagaagcgagcgcagUu..................   1   1   iD2
    .......................................................................gggagcggagaagcgagcgcag....................   1   0   iD2
    .......................................................................gggagcggagaagcgagcgcaga...................   1   0   iD2
    ........................................................................ggagcggagaagcgagcgc......................   1   0   iD2
    ........................................................................ggagcggagaagcgagcgcag....................   1   0   iD2
```

I.

Provisional ID	: chr17_8017
Score total	: 1.5
Score for star read(s)	: -1.3
Score for read counts	: 0
Score for mfe	: 2
Score for randfold	: -2.2
Score for cons. seed	: 3
Total read count	: 40
Mature read count	: 37
Loop read count	: 0
Star read count	: 3

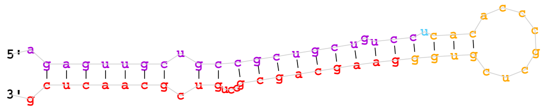

QXBT9

chr17:78140754..78140815:+

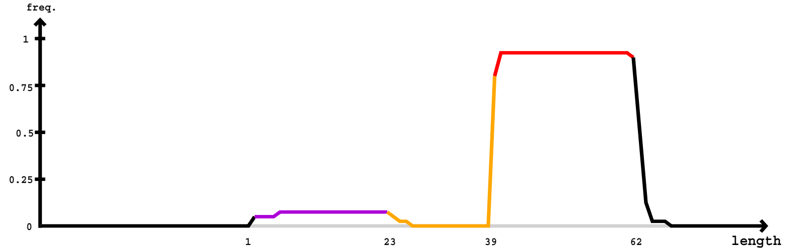

Star Mature

```
5'- cuacuuugggugcgggcuggcccaugggccgcagaguugcugccgcugcuguccucacacccgcucguggggaagcagcgccugucgcaacucgccacuuguucuccucacagc  -3'   obs
    cuacuuugggugcgggcuggcccaugggccgcagaguugcugccgcugcuguccucacacccgcucguggggaagcagcgccugucgcaacucgccacuuguucuccucacagc         exp
    ...((.((((.(.((((.((((....))))((.(((((((.(((((((((.(((.(((........)))))))))))))...)).))))))))).....)))).))))).)).     reads   mm   sample
    ................................agaguugcugccgcugcugucU...........................................................   2       1    iD7
    ....................................uugcugccgcugcuguccuca........................................................   1       0    iD7
    ......................................................................gaagcagcgccugucgcaacuc.....................   1       0    iD7
    ......................................................................gaagcGgcgccugucgcaacucg....................   1       1    iD7
    ......................................................................gaagcagcgccugucgcaacucA....................   3       1    iD7
    ......................................................................gaagcagcgccugucgcaacucU....................   1       1    iD7
    ......................................................................gGagcagcgccugucgcaacucg....................   1       1    iD7
    ......................................................................gaagcagcgccugucgcaacucg....................   20      0    iD7
    ......................................................................gaagcagcgccugucgcaacucgU...................   1       1    iD7
    ......................................................................gaagcagcgccugucgcaacucgc...................   1       0    iD7
    ......................................................................gaagcagcgccugucgcaacucgcc..................   2       0    iD7
    ......................................................................gaagcagcgccugucgcaacucgcU..................   1       1    iD7
    .......................................................................aagcagcgccugucgcaacucg....................   1       0    iD7
    .......................................................................aagcagcgccugucgcaacucgU...................   1       1    iD7
    .......................................................................aagcagcgccugucgcaacucgc...................   1       0    iD7
    .......................................................................aagcagcgccugucgcaacucgcc..................   1       0    iD7
    .......................................................................aagcagcgccugucgcaacucgccaUu...............   1       1    iD7
```

J.

Provisional ID	:	chr1_171
Score total	:	12.4
Score for star read(s)	:	3.9
Score for read counts	:	8.3
Score for mfe	:	-0.7
Score for randfold	:	1.6
Score for cons. seed	:	-0.6
Total read count	:	28
Mature read count	:	27
Loop read count	:	0
Star read count	:	1

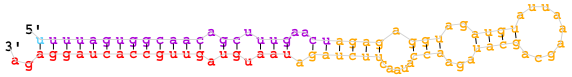

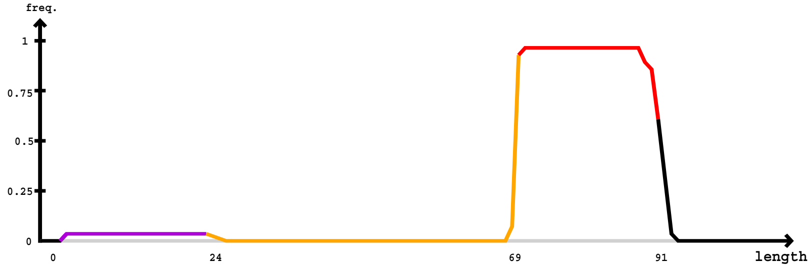

QXBT10

chr1:19910424..19910514:+

```
   Star                                                              Mature
5'- auuuuaguggcaacagcuuugaacuagagagguagauguauuaaagcagcauagaaccauaacuucuagauaauguaguugccacuaggagauauuagaaaguuagaaugu  -3'   obs
    auuuuaguggcaacagcuuugaacuagagagguagauguauuaaagcagcauagaaccauaacuucuagauaauguaguugccacuaggagauauuagaaaguuagaaugu         exp
    .((((((((((((.((.(((..((((((.(((..((((.........))))...))).....)))))).))).)).))))))))))))....................     reads   mm   sample
    ..uuuaguggcaacagcuuugaacu....................................................................................     1       0    iD7
    .....................................................................auaauguaguugccacuagg....................     2       0    iD7
    ......................................................................uaauguaguugccacuagga...................     1       0    iD7
    ......................................................................uaaCguaguugccacuaggag..................     1       1    iD7
    ......................................................................uaauguaguugccacuaggag..................     6       0    iD7
    ......................................................................uaauguaguugccacuaggagU.................     5       1    iD7
    ......................................................................uaauguaguugccacuaggaga.................     6       0    iD7
    ......................................................................uaauguaguugccacuGggaga.................     1       1    iD7
    ......................................................................uaauguaguugccacuaggaAa.................     1       1    iD7
    ......................................................................uaauguaguugccacuaggagaC................     1       1    iD7
    ......................................................................uaauguaguugccacuaggagaA................     1       1    iD7
    ......................................................................uaauguaguugccacuaggagauU...............     1       1    iD7
    .......................................................................aauguaguugccacuaggagU.................     1       1    iD7
```

K.

Provisional ID	:	chr9_16125
Score total	:	5.8
Score for star read(s)	:	-1.3
Score for read counts	:	0
Score for mfe	:	2.5
Score for randfold	:	1.6
Score for cons. seed	:	3
Total read count	:	27
Mature read count	:	26
Loop read count	:	0
Star read count	:	1

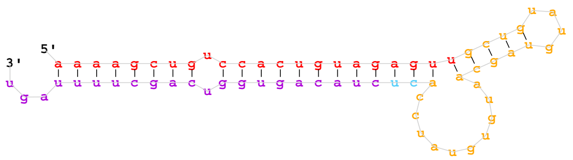

QXBT11

chr9:32456301..32456370:-

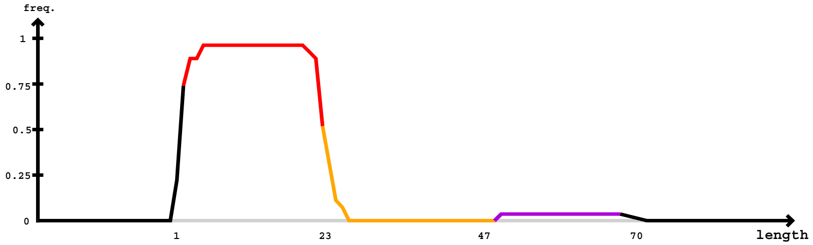

Mature **Star**

```
5'- ccauuuuuaugcuuacuacuaaaagcuguccacuguagaguugcuguauguagcaauguguauccacucuacaguggucagcuuuuaguagagagcauaaaaaugauaaaau  -3'   obs
    ccauuuuuaugcuuacuacuaaaagcuguccacuguagaguugcuguauguagcaauguguauccacucuacaguggucagcuuuuaguagagagcauaaaaaugauaaaau         exp
    .(((((((((((((.(((((((((((((.((((((((((((((((....)))))..........)))))))))))).)))))))))))).))))))))))))).......   reads  mm  sample
    ...................uaaaGgcuguccacuguagagu.......................................................................   1      1   DC7
    ...................uaaaagcuguccacuguagagu.......................................................................   2      0   DC7
    ...................uGaaagcuguccacuguagagu.......................................................................   1      1   DC7
    ...................uaaaagcuguccacuguagagC.......................................................................   2      1   DC7
    ....................aaaagcuguccacuguagag........................................................................   1      0   DC7
    ....................aaaagcuguccacuguagagu.......................................................................   2      0   DC7
    ....................aaaGgcuguccacuguagagu.......................................................................   1      1   DC7
    ....................aaaagcuguccacuguagagC.......................................................................   1      1   DC7
    ....................aaaGgcuguccacuguagaguu......................................................................   3      1   DC7
    ....................aaaagcuguccacuguagaguG......................................................................   1      1   DC7
    ....................aaaagcuguccacuguagaguu......................................................................   5      0   DC7
    .....................aGagcuguccacuguaga.........................................................................   1      1   DC7
    .....................aaagcuguccacuguagaguu......................................................................   2      0   DC7
    .....................aaagcuguccacuguagaguugU....................................................................   1      1   DC7
    .......................agcuguccacuguagaguugcu...................................................................   1      0   DC7
    .......................agcuguccacuguagaguugcC...................................................................   1      1   DC7
    ....................................................................cuacGguggucagcuuuuagu.......................   1      1   DC7
```

L.

Provisional ID	:	chr4_12297
Score total	:	0.4
Score for star read(s)	:	-1.3
Score for read counts	:	0
Score for mfe	:	0.9
Score for randfold	:	-2.2
Score for cons. seed	:	3
Total read count	:	31071
Mature read count	:	31071
Loop read count	:	0
Star read count	:	0

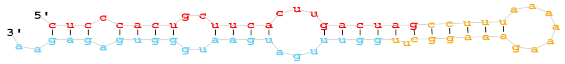

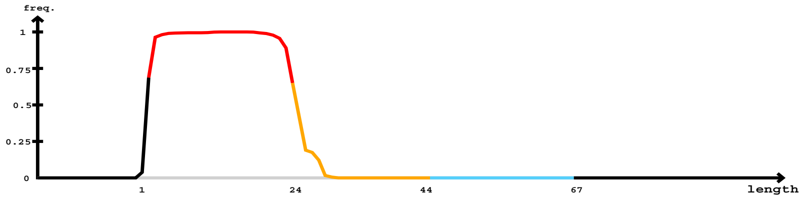

QXBT12

chr4:165400665..165400731:-

```
                 Mature                      Star
5'- uuuauuccuucuuuacucccacugcuucacuugacuagccuuuaaaaaagaaaggcuugguuugaugaaugggugagagaaaaggugaguguuagaggugaaacugcccuuc  -3'   exp
    ..(((((..(((((.(((.((((..((((...((((((((((.......)))))).)))))...))))..)))).))).))))).)))))..((.(((......)))))..   reads   mm   sample
    ........uucuuCacucccacugcuucacuugacuagccuu......................................................................   1       1    DC7
    ............uCacucccacugcuucacuugacuagccuu......................................................................   2       1    DC7
    .............uacucccacugcuucacuugacuagccuu......................................................................   1       0    DC7
    .............Aacucccacugcuucacuugacuagccuu......................................................................   1       1    DC7
    .............uacucccacugcuucacuugacuagccuC......................................................................   1       1    DC7
    ..............acucccacGgcuucacu.................................................................................   1       1    DC7
    ..............acucccacugcuucacu.................................................................................   18      0    DC7
    ..............acucccacugcuucacuu................................................................................   3       0    DC7
    ..............acucccacugcuucacuug...............................................................................   4       0    DC7
    ..............acucccacugcuucacuuga..............................................................................   5       0    DC7
    ..............acucccacugcuucacuugac.............................................................................   12      0    DC7
    ..............acucccacCgcuucacuugacu............................................................................   1       1    DC7
    ..............acucccacugcuucacuugacG............................................................................   1       1    DC7
    ..............acucccacugcuAcacuugacu............................................................................   1       1    DC7
    ..............Ccucccacugcuucacuugacu............................................................................   2       1    DC7
    ..............acucccacugcuucacuugacu............................................................................   69      0    DC7
    ..............acucccacugcuucacuugacua...........................................................................   69      0    DC7
    ..............acucccacugcuucacuugacGa...........................................................................   1       1    DC7
    ..............acucccacugcuAcacuugacuag..........................................................................   1       1    DC7
    ..............acucccacugcuucacuugacuaC..........................................................................   2       1    DC7
    ..............acucccacugcuucacuugUcuag..........................................................................   1       1    DC7
    ..............acucccacugcAucacuugacuag..........................................................................   1       1    DC7
    ..............acucccacugcuucacuugacuag..........................................................................   257     0    DC7
    ..............acucccacCgcuucacuugacuag..........................................................................   1       1    DC7
    ..............acucccacugcuucacuAgacuag..........................................................................   1       1    DC7
    ..............acucccacugcuucUcuugacuag..........................................................................   1       1    DC7
    ..............aAucccacugcuucacuugacuag..........................................................................   1       1    DC7
    ..............acAcccacugcuucacuugacuag..........................................................................   1       1    DC7
    ..............acucccacugcuucacAugacuag..........................................................................   1       1    DC7
    ..............acucccacugcuucacuugacuagc.........................................................................   54      0    DC7
    ..............acucccacugcuucacuugacuCgc.........................................................................   1       1    DC7
    ..............acucccacugcuucUcuugacuagc.........................................................................   1       1    DC7
    ..............acucccacugcuucacuugacAagcc........................................................................   1       1    DC7
    ..............acucccacugcuucacuuAacuagcc........................................................................   1       1    DC7
```

M.

Provisional ID	:	chr14_3835
Score total	:	0
Score for star read(s)	:	-1.3
Score for read counts	:	0
Score for mfe	:	0.5
Score for randfold	:	-2.2
Score for cons. seed	:	3
Total read count	:	124795
Mature read count	:	124795
Loop read count	:	0
Star read count	:	0

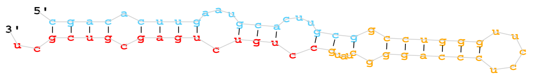

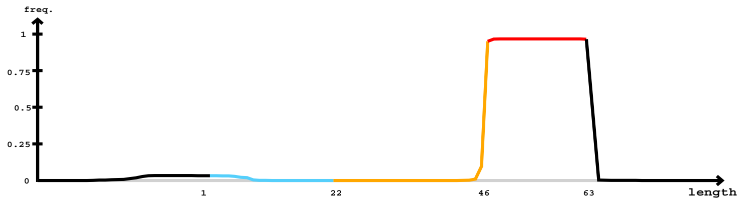

QXBT13

chr14:16057563..16057625:+

Star Mature

```
5'- ugugaacugcaggacacauugaucaucgacacuugaaugcacuugcggccuggguuccucccagggcuaugccugucugagcgucgcuugccgaucaaaauuccccagg   -3'   exp
    ......(((..(((....((((((..((((.((((...(((...(((.((((((.....)))))).)...)).))).)))).)))).......))))))...))).))).   reads   mm   sample
```

alignment	reads	mm	sample
.gugaacugcaggacacauu........	1	0	iD4
.gugaacugcaggacacauug........	1	0	iD4
.gugaacugcaggacacauugauc........	1	0	iD4
..ugaacugcaggaGacauu........	1	1	iD4
..ugaacugcaggacacauu........	2	0	iD4
..ugaacugcaggacacauugau........	1	0	iD4
...gaaAugcaggacacauu........	1	1	iD4
...gaaGugcaggacacauugauc........	1	1	iD4
....aacugcaggacacauugaucaucgacac........	1	0	iD4
....aacugcaggacacauugaucaucgacacu........	1	0	iD4
.....acugcaggacacauugaucaucg........	1	0	iD4
.....aGugcaggacacauugaucaucgac........	1	1	iD4
.....aAugcaggacacauugaucaucgaca........	1	1	iD4
.....acugUaggacacauugaucaucgaca........	1	1	iD4
.....aGugcaggacacauugaucaucgacacu........	2	1	iD4
.......ugcaggacacauugauc........	9	0	iD4
.......ugcaggacacauugauG........	1	1	iD4
.......ugcaggacacauugauU........	1	1	iD4
.......ugcaggacacauugauca........	4	0	iD4
.......ugcaggacacauugaucau........	1	0	iD4
.......ugcaggacacauugaucauc........	6	0	iD4
.......ugcaggacacauugaucauU........	1	1	iD4
.......ugcaggacacauugaucaucA........	2	1	iD4
.......ugcaggacacauugaucaucg........	3	0	iD4
.......ugcaggacacauugaucaucgaU........	1	1	iD4
.......ugcaggacacauugaucaucgac........	6	0	iD4
.......ugcaggacacauugaucaucgacU........	1	1	iD4
.......ugcaggacacauugaucaucgaca........	12	0	iD4
.......ugcaggacacauugaucaucgacacC........	1	1	iD4
.......ugcaggacacauugaucaucgacacu........	9	0	iD4
.......ugcaggacacauugaucaucgacacuu........	1	0	iD4
.......ugcaggacacauugaucaucgacacuuU........	1	1	iD4
........gcaggacacauugauca........	4	0	iD4
........gcaggacacauugaucG........	1	1	iD4

N.

Provisional ID	:	chr22_9127
Score total	:	19694.9
Score for star read(s)	:	3.9
Score for read counts	:	19693.7
Score for mfe	:	0.2
Score for randfold	:	-2.2
Score for cons. seed	:	-0.6
Total read count	:	38640
Mature read count	:	38019
Loop read count	:	7
Star read count	:	614

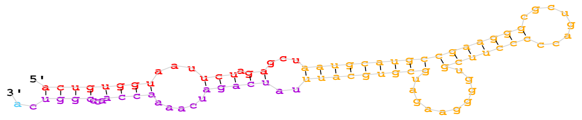

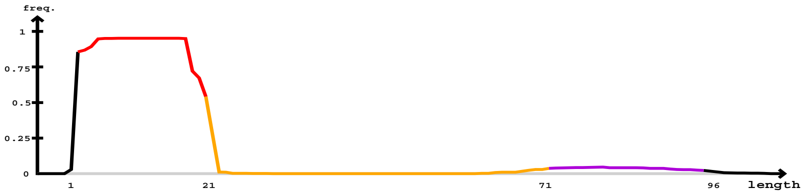

QXBT14

chr22:11629695..11629789:+

```
    Mature                                                          Star
5'- gaaaacugugguaauucuagagcuaaugcaugccgaagggcgcugaccccccuucguggggaagaugcgugcauuuaucagaucaaaaccaacccggucagccccucucu  -3'   obs
    gaaaacugugguaauucuagagcuaaugcaugccgaagggcgcugaccccccuucguggggaagaugcgugcauuuaucagaucaaaaccaacccggucagccccucucu        exp
    ....((((((((...(((.((...((((((((((((((((........)))))))..........)))))))))..)))))......))))...))))...........   reads   mm   sample
    gaUaacugugguaauucuagagcuaaugcau...............................................................................   1       1    iD2
    gaUaacugugguaauucuagagcuaaugcaugcc............................................................................   1       1    iD2
    .aCaacugugguaauucu............................................................................................   1       1    iD2
    .aCaacugugguaauucuag..........................................................................................   1       1    iD2
    .aCaacugugguaauucuagagcu......................................................................................   1       1    iD2
    .aUaacugugguaauucuagagcuaaug..................................................................................   2       1    iD2
    .aUaacugugguaauucuagagcuaaugcaugccg...........................................................................   1       1    iD2
    ..aaacugugguaauucuagagc.......................................................................................   2       0    iD2
    ..Caacugugguaauucuagagcu......................................................................................   1       1    iD2
    ..aaacugugguaauucuagagcu......................................................................................   1       0    iD2
    ..Uaacugugguaauucuagagcuaaug..................................................................................   1       1    iD2
    ..Uaacugugguaauucuagagcuaaugcaugc.............................................................................   2       1    iD2
    ..Uaacugugguaauucuagagcuaaugcaugcc............................................................................   1       1    iD2
    ...aacugugguaauucuag..........................................................................................   47      0    iD2
    ...aacugugguaauucGag..........................................................................................   1       1    iD2
    ...aacCgugguaauucuag..........................................................................................   2       1    iD2
    ...aacugugguaaCucuag..........................................................................................   1       1    iD2
    ...Uacugugguaauucuag..........................................................................................   1       1    iD2
    ...Uacugugguaauucuaga.........................................................................................   1       1    iD2
    ...aGcugugguaauucuaga.........................................................................................   1       1    iD2
    ...aacugugguaUuucuaga.........................................................................................   1       1    iD2
    ...aacugugguaauucuaAa.........................................................................................   1       1    iD2
    ...Cacugugguaauucuaga.........................................................................................   1       1    iD2
    ...aacugugguaauucuGga.........................................................................................   2       1    iD2
    ...aacugugguaauucuagG.........................................................................................   4       1    iD2
    ...aacugugguaauucCaga.........................................................................................   1       1    iD2
    ...aacugugguaauucuaga.........................................................................................   112     0    iD2
    ...aacugugguaauucuagU.........................................................................................   1       1    iD2
    ...aacugugguaauucuagag........................................................................................   91      0    iD2
    ...aacCgugguaauucuagag........................................................................................   1       1    iD2
    ...aacugugguaauCcuagag........................................................................................   1       1    iD2
    ...aacugugguaauucuagaA........................................................................................   3       1    iD2
    ...aacugugguaauucuGgag........................................................................................   1       1    iD2
```

O.

Provisional ID	: chr4_26072
Score total	: 18.3
Score for star read(s)	: 3.9
Score for read counts	: 7.8
Score for mfe	: 2
Score for randfold	: 1.6
Score for cons. seed	: 3
Total read count	: 27
Mature read count	: 24
Loop read count	: 0
Star read count	: 3

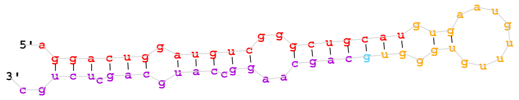

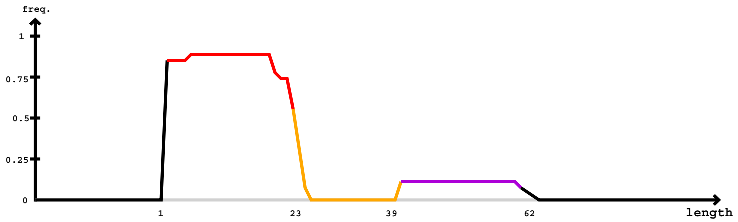

QXBT15

chr4:141707992..141708053:+

```
                      Mature                              Star
5'- uggagaggcagagcugcugcaggacuggaugucgggcugcaugugaauguuugugggugcagcaaggccaugcagcucugcaguggccuuucaugggaguagguugccacau  -3'  obs
    uggagaggcagagcugcugcaggacuggaugucgggcugcaugugaauguuugugggugcagcaaggccaugcagcucugcaguggccuuucaugggaguagguugccacau       exp
    ..((((((.....((((((((.(((.(((((..(((((((.((........)).)))))))..)).))).))).))))))))))))))).(((.(......).)))...      reads   mm   sample
    ....................aggacuggaugucgggcugcau......................................................................   2       0    DC2
    ....................aggacuggaugucgggcugcaG......................................................................   1       1    DC2

    ....................aggacuggaugucgggcu..........................................................................   2       0    DC4
    ....................aggacuggaugucggAcug.........................................................................   1       1    DC4
    ....................aggacuggaugucgggcugcG.......................................................................   1       1    DC4
    ....................aggacuggaugucgggcugcU.......................................................................   1       1    DC4
    ....................aggacuggaugucgggcugca.......................................................................   2       0    DC4
    ....................aggacuggaugucgggcugcau......................................................................   5       0    DC4
    ....................aggacuggaugucgggcugcaG......................................................................   1       1    DC4
    ....................aggacuggaugucgggcugcaA......................................................................   1       1    DC4
    ....................aggacuggaugucgggcugcauAu....................................................................   1       1    DC4
    ....................aggacuggaugucgggcugcauUu....................................................................   1       1    DC4
    ...........................................................cagcaaggccaugcagcucugc...............................   1       0    DC4
    ...........................................................cagcaaggccaugcagcucugA...............................   1       1    DC4

    ....................aggacuggaugucgggcu..........................................................................   1       0    DC7
    ....................aggacuggaugucgggcugca.......................................................................   1       0    DC7
    ........................cuggaugucgggcugUau......................................................................   1       1    DC7

    ....................aggacuggaugucgggcugcauU.....................................................................   1       1    iD7

    ....................aggacuggaugucgggcugcaG......................................................................   1       1    iD2
    ...........................................................cagcaaggccaugcagcucu.................................   1       0    iD2
```

P.

Provisional ID	: chr14_10595
Score total	: 5.3
Score for star read(s)	: -1.3
Score for read counts	: 0
Score for mfe	: 2
Score for randfold	: 1.6
Score for cons. seed	: 3
Total read count	: 16
Mature read count	: 16
Loop read count	: 0
Star read count	: 0

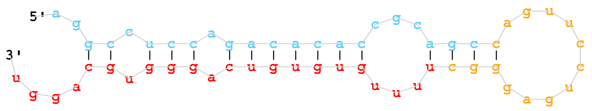

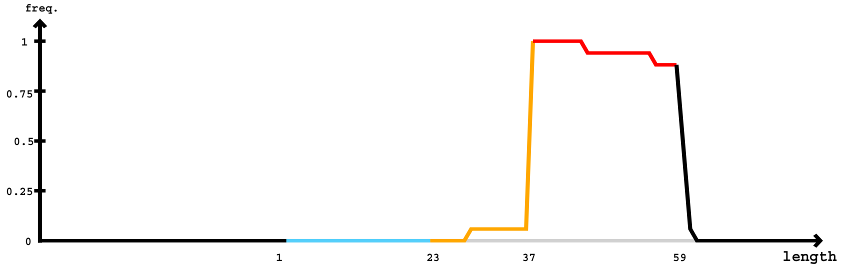

QXBT16

chr14:94113640..94113698:+

```
                                     Star                                Mature
5'- ccguaugaaugcccaucagcugagccuaacaggcaggccuccagacacaccgcagccaguuccugagggcuuuugugugucagggugcaggucugcugggcugccagccaac   -3'    exp
    ..................(((((((((.(((((..((.(((.(((((((...((((..........))))...))))))).))).))..))))).))))))..))))....   reads   mm   sample
    ......................................................................uuuugugugucagggugcaggu....................   2       0    DC2

    ......................................................................uuuugugugucagggugcaggu....................   1       0    DC7

    ......................................................................uuuugugugucagggugc........................   1       0    DC4
    ......................................................................uuuugugugucagggugcaggC....................   1       1    DC4
    ......................................................................uuuugugugucagggugcaggu....................   7       0    DC4
    ......................................................................uuuugugugucagggugcaggA....................   1       1    DC4
    ......................................................................uuuugugugucagggugcagguA...................   2       1    DC4
    ......................................................................uuuugugugucagggugcagguAu..................   1       1    DC4

    .............................................................ccugagggcuuuuCugu..................................   1       1    iD2
```

Q.

Provisional ID	:	chr2_19753
Score total	:	3.1
Score for star read(s)	:	-1.3
Score for read counts	:	-2.3
Score for mfe	:	2.2
Score for randfold	:	1.6
Score for cons. seed	:	3
Total read count	:	7
Mature read count	:	6
Loop read count	:	1
Star read count	:	0

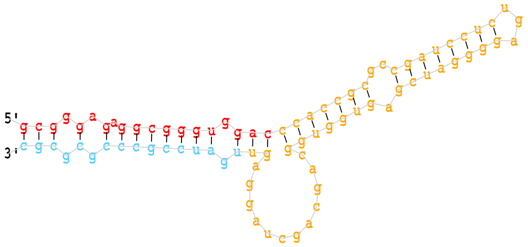

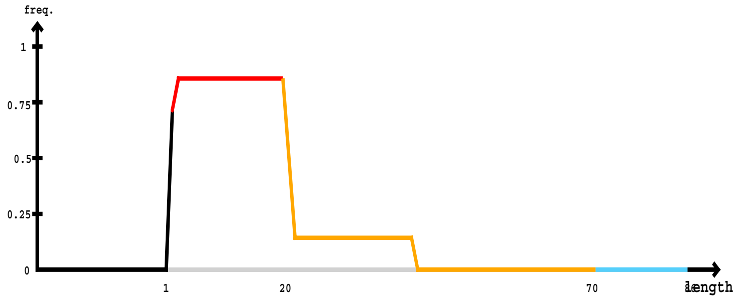

QXBT17

chr2:136117376..136117461:-

```
                    Mature                                        Star
5'- cccagcacaguaggcagagggcgggagaggcggguggacccaccgcgccgauccucugaggggaucgagugguggcagcagcuaggaguugauccgcccgcgcgcuuug   -3'    exp
    .............(((((.(((.(...(((((((.((((((((((..((((((((...)))))))).)))))))...........))).)))))))).))).)))))   reads   mm   sample
    ....................gcgggagaggcggguggac......................................................................   2       0    DC2

    ....................gcgggagaggcggguggaU......................................................................   1       1    DC4
    ....................gcgggagaggcggguggac......................................................................   2       0    DC4

    ........................................caccgcgccgauccucugag.................................................   1       0    iD7

    .....................cgggagaggcggguggaG......................................................................   1       1    iD2
```

R.

Provisional ID	:	chrX_38905
Score total	:	2.1
Score for star read(s)	:	-1.3
Score for read counts	:	0
Score for mfe	:	2.4
Score for randfold	:	1.6
Score for cons. seed	:	-0.6
Total read count	:	39
Mature read count	:	39
Loop read count	:	0
Star read count	:	0

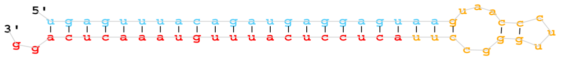

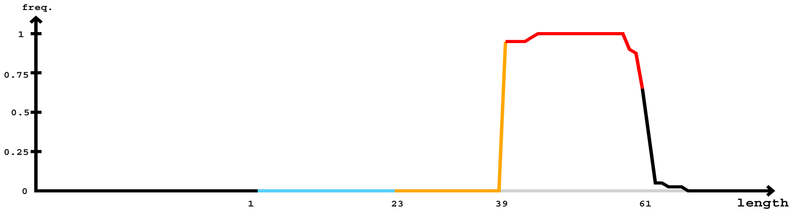

QXBT18

chrX:129790441..129790501:+

Star Mature

```
5'- gccuugggacuugaaccugaauucccuuggccugaguuuacagaugaggaguaaguaacccuugggccuuacuccucauuuguaaacucaggccaacaauaccacuuaccu  -3'   exp
    .....((((.((.......)).))))((((((((((((((((((((((((((((...((...))..))))))))))))))))))))))))))))................   reads   mm   sample
    .....................................................................acuccucauuuguaaacucagg...................   1       0    DC2

    .....................................................................acuccucauuuguaaacucag....................   2       0    DC7
    .....................................................................acuccucauuuguaaacucagg...................   1       0    DC7

    .....................................................................acuccucauuuguaaacuc......................   3       0    DC4
    .....................................................................acuccucauuuguaaacucagC...................   1       1    DC4
    .....................................................................acuccucauuuguaaacucagg...................   1       0    DC4
    .....................................................................acuccucauuuguaaacucagA...................   1       1    DC4

    .....................................................................acuccucauuuguaaacuU......................   1       1    iD7
    .....................................................................acuccucauuuguaaacuca.....................   1       0    iD7
    .....................................................................acuccucauuuguaaacucag....................   2       0    iD7
    .....................................................................acuccucauuuguaaacucagg...................   9       0    iD7
    .....................................................................acuccucauuugGaaacucagg...................   1       1    iD7
    .....................................................................acuccucauuuguaaacucagU...................   1       1    iD7
    .........................................................................cucauuuguaaacucaggcca................   1       0    iD7
    ..........................................................................ucauuuguaaacucaggccaaUa.............   1       1    iD7

    .....................................................................acuccCcauuuguaaacucag....................   1       1    iD2
    .....................................................................acuccucauuuguaaacucag....................   2       0    iD2
    .....................................................................acuccucauuuguaaacucagA...................   1       1    iD2
    .....................................................................acuccucauuuguaaacucagg...................   3       0    iD2

    .....................................................................acuccucauuuguaaacucag....................   1       0    iD4
    .....................................................................acuccucauuuguaaacucaA....................   1       1    iD4
    .....................................................................acuccucauuuguaaacucagg...................   1       0    iD4
    .....................................................................acuccucauuuguaaacucagU...................   2       1    iD4
    .....................................................................acuccuUauuuguaaacucagg...................   1       1    iD4
```

S.

Provisional ID	: chr8_12884
Score total	: 2.1
Score for star read(s)	: -1.3
Score for read counts	: 0
Score for mfe	: 2.4
Score for randfold	: 1.6
Score for cons. seed	: -0.6
Total read count	: 171
Mature read count	: 171
Loop read count	: 0
Star read count	: 0

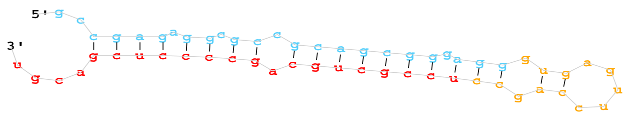

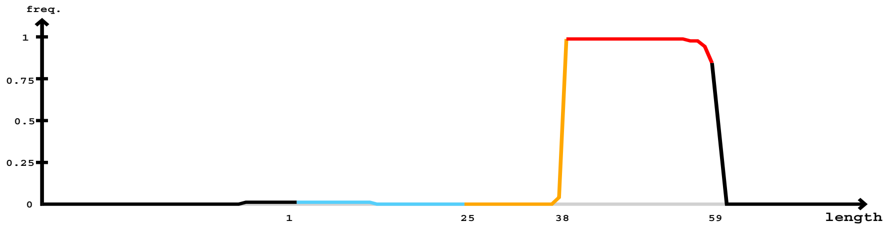

QXBT19

chr8:55880664..55880722:-

```
                                          Star                                  Mature
5'- agccggcaaaagcaccggacugcuccucggcaggccgagaggcgccgcagcgggaggggugaguuccagccuccgcugcagccccucgacgucacgcuccugcccggggcca  -3'    exp
    ((((((........)))).))(((((..((((((.((((.((.((.(((((((.(((.((.....)).))))))))))).))))))))..........)))))).)))))..      reads   mm    sample
    ..........................ucggcaggccgagaggcg....................................................................      2       0     iD4
    ......................................................................cuccgcugcagccccucgac......................      1       0     iD4
    ......................................................................cuccgcugcagccccucgacg.....................      1       0     iD4
    ......................................................................cuccgcugcagccccucgacgu....................      5       0     iD4
    .......................................................................uccgcugcagccccucg........................      1       0     iD4
    .......................................................................uccgcugcagccccucA........................      1       1     iD4
    .......................................................................uccgcugcagccccucgaU......................      1       1     iD4
    .......................................................................uccgcugcagccccucgac......................      4       0     iD4
    .......................................................................uccgcugcagccccucgacg.....................      11      0     iD4
    .......................................................................uccgcugcagccccucgacC.....................      1       1     iD4
    .......................................................................uccgcugcagccccucgacU.....................      2       1     iD4
    .......................................................................uccgcugcagccccucgacA.....................      2       1     iD4
    .......................................................................uccgcugcagccUcucgacgu....................      1       1     iD4
    .......................................................................uccUcugcagccccucgacgu....................      1       1     iD4
    .......................................................................uccgcugcagUcccucgacgu....................      1       1     iD4
    .......................................................................uccgcugcagccccucgacgC....................      24      1     iD4
    .......................................................................uccgcugcagccccucgacgG....................      1       1     iD4
    .......................................................................uccgcugUagccccucgacgu....................      1       1     iD4
    .......................................................................uccgcugcagccccucgacgu....................      87      0     iD4
    .......................................................................uccgcugcagccccucgacUu....................      1       1     iD4
    .......................................................................uccgcugcagccccucgacgA....................      4       1     iD4
    .......................................................................uccgUugcagccccucgacgu....................      1       1     iD4
    .......................................................................uccgcugcagccccucgacguA...................      4       1     iD4
    .......................................................................uccgcugcagccccucgacguU...................      10      1     iD4
    .......................................................................uccgcugcagccccucgacguc...................      5       0     iD4
```

T.

Provisional ID	: chr17_7803
Score total	: 1.3
Score for star read(s)	: -1.3
Score for read counts	: 0
Score for mfe	: 1.6
Score for randfold	: 1.6
Score for cons. seed	: -0.6
Total read count	: 18
Mature read count	: 18
Loop read count	: 0
Star read count	: 0

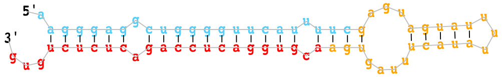

QXBT20

chr17:42311190..42311260:+

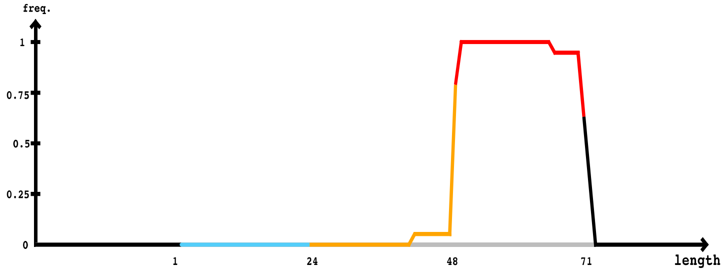

Star **Mature**

```
5'- gauguaaccgauucaucucucagaagggaggcuggggguucauuuucgaguaguauuuuauacuuuagugaacguggacuccagacucucugugaacccuaugagagcgcgucu  -3'    exp
    (((((...........((((((..((((((.(((((((((((.((((...(((((...)))))....)))).)))))))))))).)))))).........)))))).))))).  reads   mm    sample
    ................................................................uuagugaacguggacuccagacuc..........................  1       0     iD7
    .......................................................................acguggacuccagacucucuUu.....................  2       1     iD7
    .......................................................................acguggacuccagacucucugu.....................  2       0     iD7
    .......................................................................acguggacuccagacucucuguA....................  1       1     iD7
    .......................................................................acguggacuccagacucucuUug....................  1       1     iD7
    .......................................................................acguggacuccagacucucugug....................  5       0     iD7
    .......................................................................acguggacuccagacucucugugU...................  1       1     iD7
    .......................................................................acguggacuccagacucucuguga...................  2       0     iD7
    ........................................................................cguggacuccagacucucugu.....................  2       0     iD7
    ........................................................................cguggacuccagacucucugug....................  1       0     iD7
    ........................................................................cguggacuccagacucucugugU...................  1       1     iD7
```

U.

Provisional ID	: chr17_5067	
Score total	:	22.9
Score for star read(s)	:	3.9
Score for read counts	:	12.4
Score for mfe	:	2
Score for randfold	:	1.6
Score for cons. seed	:	3
Total read count	:	36
Mature read count	:	32
Loop read count	:	0
Star read count	:	4

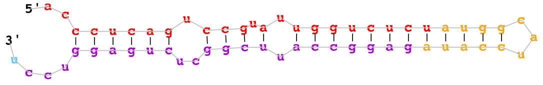

QXBT21

chr17:7306830..7306888:−

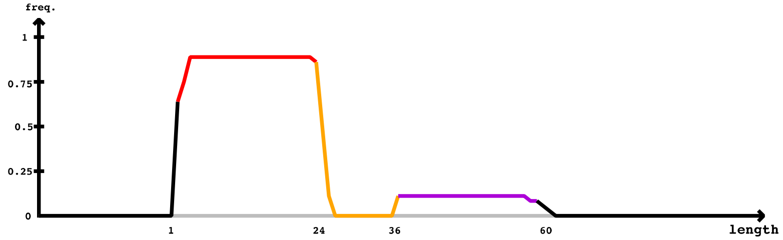

Mature **Star**

```
5'- gcggacaccaucuucuuuaaacccucaguccguauuggucucuauggcauccauagaggccauucggcucugagguccucaguaaagaaacuuagaugguauuacuguguuuu  -3'   obs
    gcggacaccaucuucuuuaaacccucaguccguauuggucucuauggcauccauagaggccauucggcucugagguccucaguaaagaaacuuagaugguauuacuguguuuu        exp
    ((((..(((((((((((((...((((((.(((.(.((((((((((((...)))))))))))).))))..)))))).......)))))).....)))))))....)))).....   reads   mm   sample
    ....................acccucaguccguauuggucuc.......................................................................   1       0    DC4
    ....................acccucaguccguauuggucucA......................................................................   1       1    DC4
    ....................acccucaguccguauuggucucu......................................................................   10      0    DC4
    ....................acccucaguccguauuggucucC......................................................................   8       1    DC4
    ....................acccucaguccguauuggucucua.....................................................................   1       0    DC4
    ....................acccucaguccguauuggucucuC.....................................................................   1       1    DC4
    ....................acccucaguccguauuggucucuUu....................................................................   1       1    DC4
    .....................cccCcaguccguauuggucucu......................................................................   1       1    DC4
    .....................cccucaguccguauuggucucu......................................................................   2       0    DC4
    .....................cccucaguccguauuggucucua.....................................................................   1       0    DC4
    ......................ccucaguccguauuggucucu......................................................................   1       0    DC4
    ......................ccucGguccguauuggucucu......................................................................   1       1    DC4
    ......................ccucaguccguauuggucucuau....................................................................   3       0    DC4
    .......................................................gaggccauucggcucugagUu.....................................   1       1    DC4
    .......................................................gaggccauucggcucugaggucc...................................   1       0    DC4
    .......................................................gaggccauucggcucugaggucU...................................   1       1    DC4
    .......................................................gaggccauucggcucugagguccu..................................   1       0    DC4
```

V.

Provisional ID	: chr22_7796
Score total	: 9.4
Score for star read(s)	: 3.9
Score for read counts	: 6.8
Score for mfe	: 1.5
Score for randfold	: -2.2
Score for cons. seed	: -0.6
Total read count	: 25
Mature read count	: 18
Loop read count	: 0
Star read count	: 7

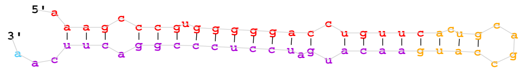

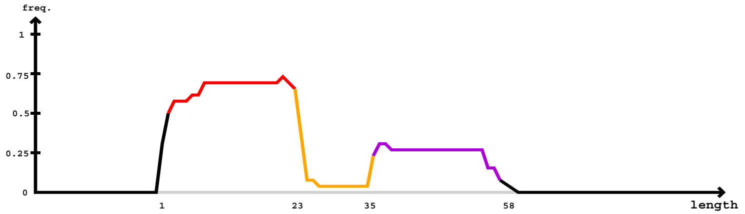

QXBT22

chr22:17108635..17108691:+

```
                Mature                      Star
5'- cggcugcgcugcgaccacggaaagcccgugggggaccuguucacugcagccaugaacaugauccucccggacuucaagaggccagccugcuucggcaccuacguagucugcu  -3'   obs
    cggcugcgcugcgaccacggaaagcccgugggggaccuguucacugcagccaugaacaugauccucccggacuucaagaggccagccugcuucggcaccuacguagucugcu        exp
    .((((((((((......))).(((.(((.(((((((.((((((.((....)))))))).).))))))))).)))....(((...(((......))).))).)))))))....   reads   mm   sample
    ...................gaaagcccgugggggaccugu........................................................................   1       0    DC4
    ...................gaaagcccgugggggaccuguu.......................................................................   1       0    DC4
    ...................gaaagcccgugggggaccuguuc......................................................................   2       0    DC4
    ...................gaaagcccgugggggaccuguuA.....................................................................   2       1    DC4
    ...................gaaagcccgugggggaccuguucU....................................................................   2       1    DC4
    ....................aaagcccgugggggaccuguuc......................................................................   4       0    DC4
    ....................aaagcccgugggggaccuguuca.....................................................................   1       0    DC4
    .....................aagcccgugggggaccuguuc......................................................................   1       0    DC4
    .....................Gagcccgugggggaccuguucacu...................................................................   1       1    DC4
    ........................cccgugggggaccuguuU......................................................................   1       1    DC4
    ..........................cgugggggaccuguuca.....................................................................   2       0    DC4
    .......................................uucacugcaAccaugaac.......................................................   1       1    DC4
    ......................................................aacaugauccucccggacC.......................................   1       1    DC4
    ......................................................aacaugauccucccggacuuc.....................................   1       0    DC4
    ......................................................aacaugauccucccggacuuU.....................................   1       1    DC4
    ......................................................aacaugauccucccggacuucC....................................   1       1    DC4
    ......................................................aacaugauccucccggacuuca....................................   1       0    DC4
    .......................................................acaugauccucccggacu.......................................   2       0    DC4
```

Figure S4. Twenty-two novel miRNA structures and reads mapping graphs (A-V) from a miRDeep2 analysis. For each novel miRNA graph, the upper left table gives the miRDeep2 score break-down for the reported miRNA. The upper right figure shows the predicted miRNA hairpin structure. The middle density plot shows the distribution of reads in the predicted precursor sequence. The sequences below indicate reads align to the precursor with the positions of the mature, loop and star strand. The red color denotes the mature sequence; the yellow color denotes the loop sequence and the purple color denotes the star sequence.

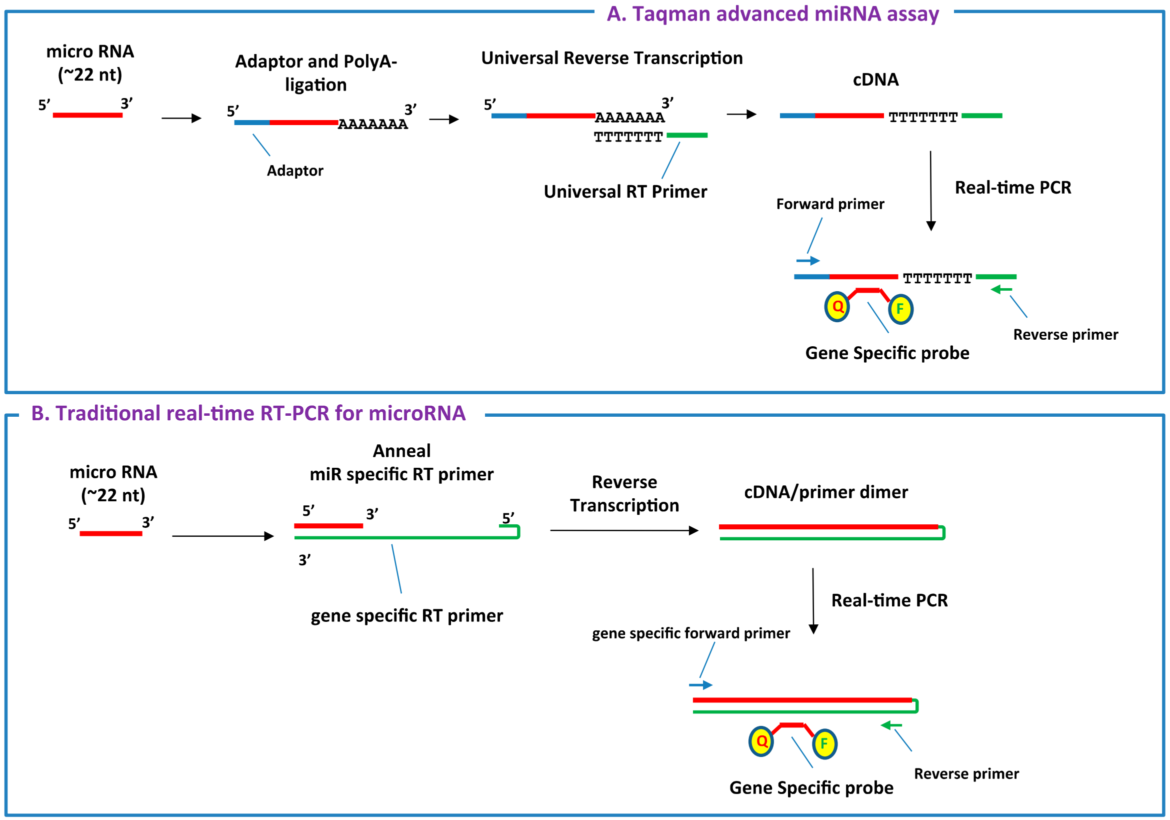

Figure S5. The schematic diagram for advanced and traditional microRNA (miRNA) qPCR assays. (**A**) In the advanced miRNA assay, the universal RT primer (containing ploy(T)) binds to the polyA tail to synthesize cDNA. (**B**) In the traditional miRNA assay, the miR specific RT primer binds to miRNA to form cDNA/primer dimer.

Table S1. Summary of sequenced immature dendritic cells (iDCs) and IL-27-treated iDCs (27DCs) small RNA library.

Sample ID	Yield (M bases)	Number of raw reads	Number of clean reads	Percentage of clean reads (%)	Number of mapped reads	Percentage of mapped reads (%)
iDC_2	2968	58182414	24040126	41.32	23200067	96.51
iDC_4	2918	57207937	27830481	48.65	27209972	97.77
iDC_7	3503	68673789	34474974	50.20	33435206	96.98
27DC_2	2513	49259191	21794678	44.24	21211384	97.32
27DC_4	2624	51456830	27426267	53.30	26484335	96.57
27DC_7	3224	63214830	24401275	38.60	23652580	96.93
